# No Reason to Worry About German Mortgages? An Analysis of Macroeconomic and Individual Drivers of Credit Risk

**DOI:** 10.1007/s10693-023-00409-3

**Published:** 2023-06-07

**Authors:** Nataliya Barasinska, Philipp Haenle, Anne Koban, Alexander Schmidt

**Affiliations:** 1 Deutsche Bundesbank, Wilhelm-Epstein-Straße 14, 60431 Frankfurt am Main, Germany; 2 European Central Bank, Sonnemannstrasse 22, 60314 Frankfurt am Main, Germany

**Keywords:** Residential real estate, Mortgages, Credit risk, Stress testing, Germany, G01, G17, G21, G28

## Abstract

This paper analyzes the macroeconomic and borrower-specific credit risk factors of residential real estate mortgages in Germany. Relying on a macroeconomic panel VAR model, we show a significant link between foreclosures, house price dynamics and unemployment. Using microeconomic regressions, we show that defaults are driven mostly by income and liquidity rather than loan-to-value (LTV) ratios. Based on those insights, we calibrate a structural model which predicts a significant increase in mortgage losses in a stress scenario, driven only partially by high-LTV loans. Hence, from a macroprudential perspective our findings support the need for a broad toolkit going beyond LTV-limits.

**Supplementary Information:**

The online version contains supplementary material available at 10.1007/s10693-023-00409-3.

## Introduction

The great financial crisis has highlighted that housing markets can pose significant risks to financial stability. Housing price booms fueled by excessive mortgage credit growth and a deterioration of credit standards contributed to a buildup of systemic risks not only in the United States but also in several European countries. Subsequently, when house prices were falling and this risk materialized, large credit losses destabilized the financial system and entailed immense negative spillovers to the real economy. Although imprudent securitization and cross-border trading in such products were important channels of risk propagation in the national and the global financial systems, the national banking sectors were mostly affected by housing downturns due to the large direct exposures of banks to residential real estate (Goodhart and Hofmann 2007).

In contrast to several other countries, the housing market in Germany has not experienced a full-blown bust during the great financial crisis as residential real estate (RRE) price and credit stock growth rates remained close to zero. In 2010 residential real estate prices and mortgages volumes started to grow again and have remained on an upward trajectory since then. Even in the recent economic downturn induced by the COVID-19 pandemic, the RRE market in Germany has so far proven robust. Despite the ongoing pandemic, by mid-2020 the RRE price and credit growth dynamics did not show any signs of deceleration. Although institutional features of the German residential real estate market are generally considered to contribute to its stability, they do not make it immune to adverse macroeconomic shocks. In the past, economic stress episodes have led to a significant increase in credit risk in banks’ residential mortgage portfolios. For instance, at the beginning of the millennium the loss rates in German RRE mortgage portfolios more than tripled, as a major macroeconomic shock triggered by the burst of the dot-com bubble hit the German economy which was already weakened by structurally high unemployment rates and weak house price development.

While the data basis remains fragmented, there are now various indications that the ongoing house price inflation trend exhibits increasing upward pressure on loan-to-value (LTV) at origination as borrowers need to finance higher acquisition prices. In particular an increasing share of loans with LTVs at origination above 100% can be observed. At the same time, a loosening of lending standards, including a relaxation of LTV constraints, has been identified as an important component of earlier international housing bubbles and busts (e.g. Cerutti et al. 2017).

Yet, up to now a comprehensive analysis of credit risk drivers for RRE lending in Germany has been hindered by the lack of detailed data on LTV ratios and other lending standards. This data gap was considered so relevant that it was pointed out in a public warning issued by the European Systemic Risk Board (ESRB) to Germany as an obstacle to the identification of risks to financial stability.^1^ This paper makes a first attempt at closing these analytical gaps and contributes to an improved understanding of RRE-related risks in Germany in various dimensions.

First, we estimate a regional panel VAR model in order to demonstrate the macroeconomic link between foreclosure rates, house price dynamics and the unemployment rate in Germany. The results confirm the view that the favorable macroeconomic environment is supporting the low default and foreclosure rates currently observable in Germany. At the same time, the results also suggest that the RRE credit performance is not immune to macroeconomic recessions and that a significant increase in default rates can be expected if unemployment surges and house price growth undergoes a sharp reversal.

Second, we run additional microeconomic regressions on German household data to shed light on which borrower characteristics can explain future credit performance. Our results suggest that mainly income and financial wealth metrics explain default rates in Germany. This contrasts with studies in the Anglo-American context, which often focus on the role of LTV ratios as a “strategic” default driver.

Third, we calibrate a structural model to project credit losses in the RRE mortgage portfolios for the entire German banking sector, explicitly accounting for past changes in house prices as well as historical LTV ratios at origination. Moreover, we take into account structural features of the German housing market and other important aspects of RRE lending in Germany. In the event of a surge in unemployment and a strong reversal of house price growth, our model predicts a significant increase in mortgage losses. While it is straightforward to show that high-LTV mortgages will generally make a disproportionately large contribution to losses, our results indicate that the more significant losses are only realized once lower LTV mortgages are affected as well. As such, it appears unlikely that high initial loan-to-value (ILTV) mortgages are solely responsible for causing a systemic RRE crisis.

Fourth, the obtained bank-by-bank loss projections can ultimately be used to assess system-wide first-round stress effects in the German banking sector. We thus contribute to understanding the extent to which the housing boom in Germany in recent years contributed to a buildup of systemic risks in the banking sector.

It is important to note, however, that we focus our analysis on RRE lending to private households but exclude RRE mortgages to enterprises as well as other commercial real estate (CRE) loans which are driven by different risk transmission mechanisms.

The reminder of the paper is organized as follows. In the next section we present stylized facts about the German housing and mortgage markets and discuss their characteristic institutional features. We subsequently discuss the empirical literature in the field of mortgage credit risk in Section 3. Sections 4 and 5 present our analytical framework and the underlying data. We discuss our results in Section 6, while Section 7 derives related (macroprudential) policy implications. Finally, Section 8 concludes.

## Stylized facts about the German housing market

### Historical performance

**Fig. 1:**
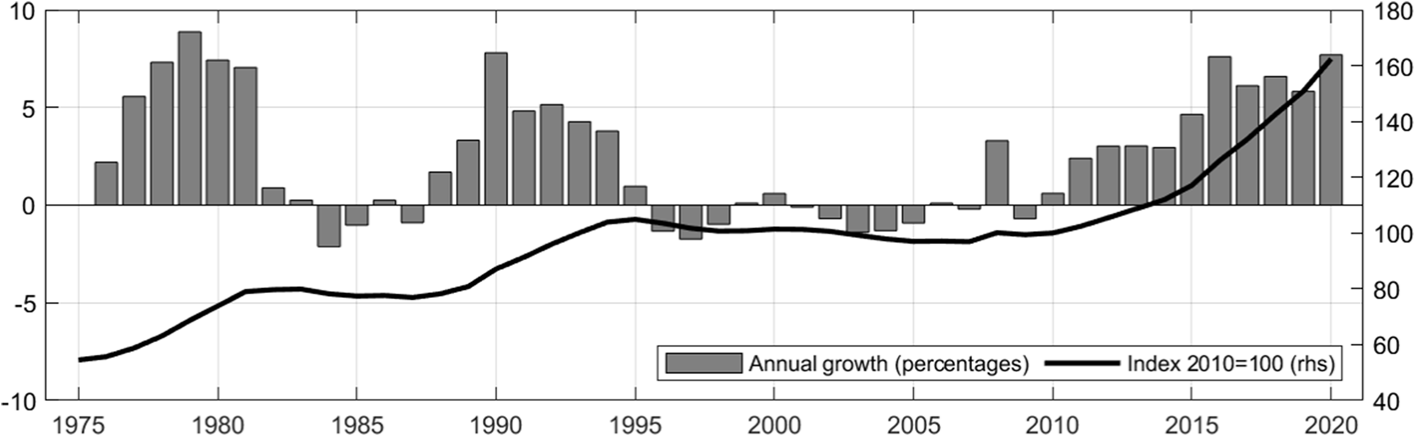
Development of house prices in Germany since 1975 according to the long time series of the Deutsche Bundesbank

In contrast to several other countries, where residential real estate markets have experienced strong upswings in the 2000 s, house prices in Germany have remained relatively flat in nominal terms during that time, implying real house price decreases (see Fig. 1).^2^ The previous German housing market boom dates back to the early 1990 s following the German reunification. However, in 2010 prices started to go back up. Since then house prices in Germany have increased by 4.6% p.a. on average up until the end of 2020 with particularly strong increases since 2016. While starting in the urban areas, the price boom spread gradually to non-urban areas and the price dynamics converged by 2019 (see Deutsche Bundesbank 2020b). These developments do not necessarily present a risk to financial stability. As Koetter and Poghosyan (2010) point out, an important indicator of systemic risk is the deviation of the price development from the respective fundamentals.

Looking at the recent price developments in Germany, fundamental factors can explain some part of the price increases. The favorable macroeconomic conditions prevailing in recent years, with robust labor markets and low interest rates as well as a structurally supported demand overhang in the housing market, contributed to the robust RRE price growth in Germany over the last decade. But historically low interest rates and a flight to assets perceived as relatively safe have also contributed to the upswing. In particular in urban areas, valuations appear to be stretched and the Bundesbank’s estimations indicate overvaluations of 15% to 30% for residential properties in urban areas in 2020 (Deutsche Bundesbank, 2021).^3^

**Fig. 2:**
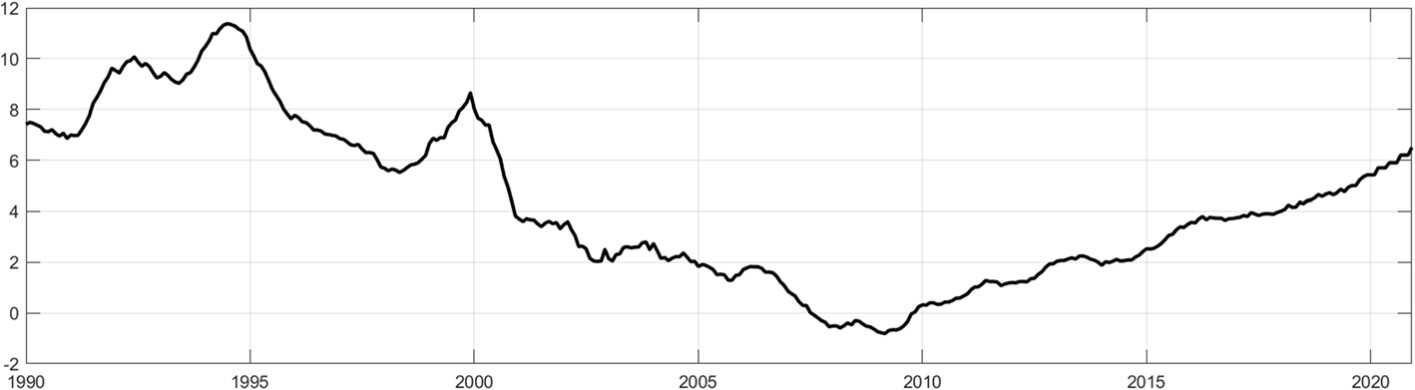
Growth of German banks’ loans to private households (including non-profit organisations) for house purchase (annual percentage changes). Source: www.bundesbank.de

This housing price inflation has been accompanied by accelerating credit dynamics with nominal annual growth of loans to households for house purchase increasing from less than 1% p.a. in 2010 to 6.5% p.a. by the end of 2020 (see Fig. 2).^4^ Also, the overall size of exposures is significant. In 2020, there were about EUR 1.4 trillion loans to German households for house purchase outstanding, which accounted for about 47% of the total credit exposure to domestic firms and households. Importantly, RRE mortgage loans are highly relevant across all banking groups and account for roughly 40% of total loans to domestic non-banks in the group of large private banks and for 50% in the group of saving banks and credit cooperatives. In contrast, the volume of residential real estate mortgage loans to enterprises was only EUR 146 billion while other CRE mortgages were amounted to 284 billion.^5^ Hence, the RRE mortgage exposure of German banks related to loans to households is much larger compared to real estate mortgage loans to domestic firms. By the end of 2020, RRE mortgage loans to households accounted for 77% of all real estate mortgage loans by German banks to domestic non-financial firms and households.

**Fig. 3:**
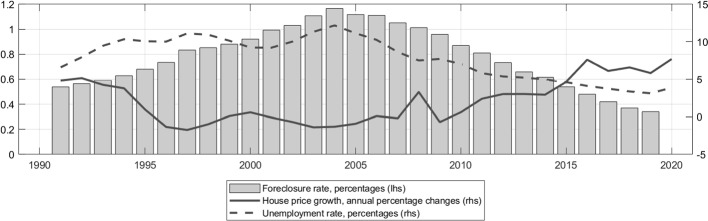
Historical unemployment rate, foreclosure rate and house price growth in Germany. Own calculations based on the long time series of the Deutsche Bundesbank and data of the Federal Statistical Office and the Federal Employment Agency

Against the background of strong house price dynamics and falling unemployment rates in the recent years, the aggregate foreclosure rate has continuously declined, reaching in 2019 its historic low of 0.35% since 1991 (see Fig. 3). This trend is mirrored by the fall in provisioning rates (see Fig. 10), suggesting decreasing levels of credit risk in the mortgage portfolios.

Finally, the German RRE market has continued to prove robust even in the light of the unprecedented economic downturn induced by the COVID-19 pandemic. While housing prices and credit stock remained on an upward trend in 2020, defaults on mortgage loans have not increased (Deutsche Bundesbank 2020a). The developments appear at first striking especially considering the significant increase in unemployment: The unemployment rate rose by 1 percentage point (pp) from 3.4% in January 2020 to 4.4% in January 2021.^6^ However, the implications of this labor market shock vary across income groups: in fact, higher-income households - which make the majority of the mortgage borrowers - suffered less from rising unemployment thanks to short-time working schemes (Deutsche Bundesbank 2020a). The benefits associated with these schemes have largely compensated for the reduction in labor income for this group of households. Hence, the liquidity position of the relevant group of households remained stable, which supported the demand for new housing loans and mitigated the default risks for the existing loans.

Hence, despite the currently low observed aggregate foreclosure rates, the large size of real estate-related exposures and significant RRE price increases in recent years call for a close monitoring of the real estate market, mortgage lending and the credit risk of underlying portfolios.

### Structural features

The German housing market is characterized by a relatively low home ownership rate of 50% and a strong rental market.^7^ Nevertheless, mortgage debt is of high importance for household balance sheets, accounting for three quarters of total household debt. Interest payments on mortgages are only tax deductible for buy-to-let properties, but not for owner-occupied real estate. Equally important is the full recourse structure of mortgages in Germany. First, this should increase the amount of assets available to satisfy the claims of the bank, hence lowering loss-given-default (LGD). Second, collateral values are less likely to act as a trigger of strategic defaults in the event of negative home equity, hence contributing to lower probabilities of default (PD) as well. Another related aspect is the average duration of legal foreclosure procedures, as longer foreclosure procedures are potentially associated with a higher cost of debt recovery. For Germany, the average time needed to complete the process was about 10 months in 2007, which is relatively short compared to the European average of just over 2 years according to European Systemic Risk Board (2015).^8^

Regarding macroprudential measures, so far, there are no regulatory requirements or limitations in Germany. In 2015, the German Financial Stability Committee recommended that the Federal Government creates a legal basis for restrictions on the LTV, the debt-service-to-income (DSTI) and debt-to-income (DTI) ratios as well as amortization requirements of RRE mortgages.^9^ The legal act that entered into force created only two of the recommended macroprudential tools: the LTV cap and the amortization requirement.^10^ These two instruments can be activated when deemed necessary to safeguard financial stability. In contrast, the income-based tools were not included in the adopted law and, hence, no restrictions on DTI or DSTI can be imposed at present.

**Fig. 4:**
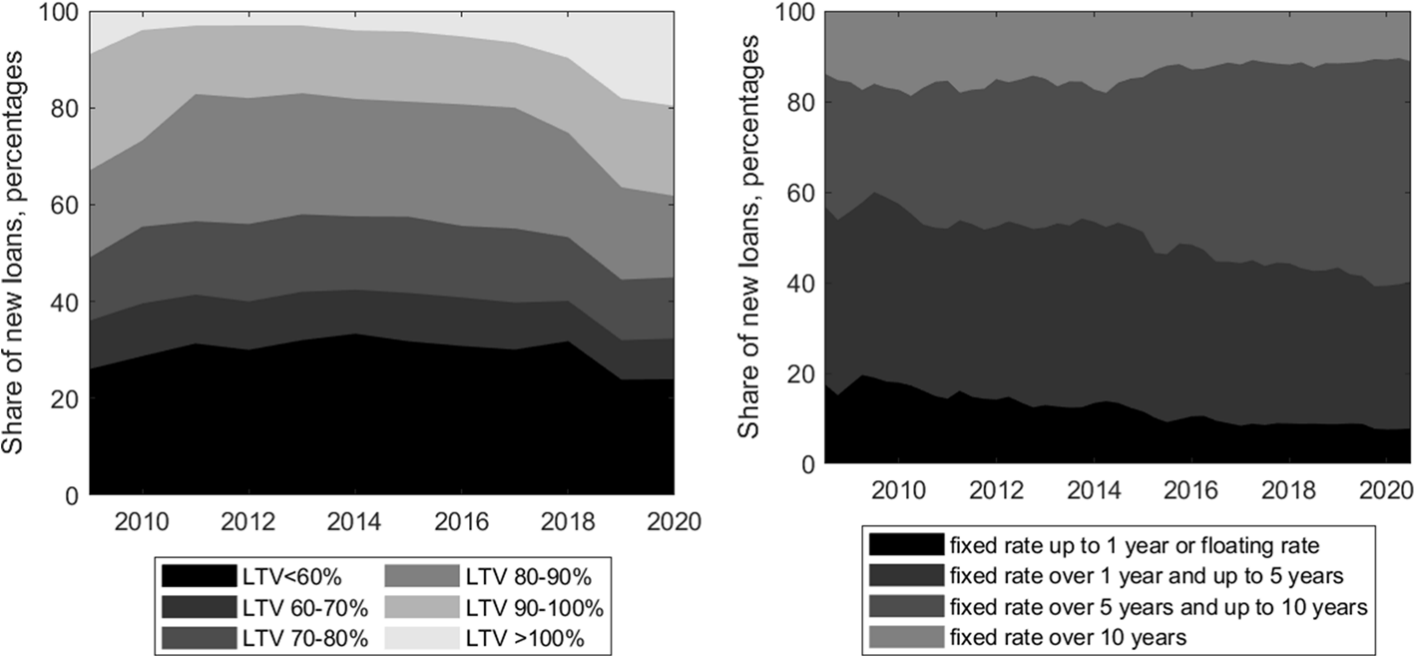
Historical distribution of new lending volume by loan-to-value ratio (left) and interest rate fixation period (right) based on the Baufinanzierungs-Index of Europace AG and the Deutsche Bundesbank

As to the recent development of LTVs in Germany, the historical distribution of new mortgages procured at the market platform Europace suggest that the majority of loans in the time period 2009-2020 had LTVs below 100% (see Fig. 4 left), although the share of high-LTV mortgages has been steadily increasing since 2017.^11^ Based on the Europace data, the average LTV was in the range 75%-80% up until 2016 and has been increasing since then, reaching the 83% on average in 2020.^12^ While these LTV figures are not necessarily representative of the whole German mortgage market, at EUR 90 billion of new loans in 2020, Europace’s market share was about 33%. In addition, the figures correspond well with estimates from other data sources. According to the Association of German Pfandbrief Banks (vdp) the average LTV was in the range of 75-79% over the time period 2009 to 2017 and increased to 82% in 2019.^13^ Finally, these figures are broadly in line with supervisory survey data on the lending practices in 2016-2018 according to which the average LTV ratio at origination increased from 82% to 84% in the respective period (Deutsche Bundesbank 2019).

Considering interest rate risk as a potential driver of defaults, it is less relevant for households in Germany compared to other countries as contracts usually have interest rate fixation periods of medium length. Around 80% of loans for house purchase in total new lending including prolongations have a fixed interest period of more than 5 years (see Fig. 4 right). Over the last years, the share of new loans with an interest rate fixation of more than 10 years has increased from around 30% to over 40%. Hence, given the predominance of the medium-length interest rate fixation periods, most existing mortgage contracts should not be immediately affected by changes in interest rates.^14^

The existing literature on institutional features of mortgage markets has focused on the Anglo-American sphere (or broader continental Europe) when explaining past booms and busts in mortgage lending. Much attention is devoted to the securitization market and its role in the US subprime mortgage crisis. Yet, in contrast to the US, securitizations play only a minor role in Germany. The volume of outstanding German mortgage-backed securities (including both RMBS and CMBS) was roughly EUR 4 billion in Q2 2019.^15^ By contrast, there were about $10 trillion worth of outstanding US mortgage-related securities by mid-2019,^16^ while the total outstanding mortgage debt was about $16 trillion (of which roughly $13 trillion for residential properties).^17^ More common than mortgage-backed securities is the refinancing of mortgage loans via German covered bonds (“Pfandbriefe”). However, this should be mainly seen as a refinancing tool rather than a credit risk transfer as banks remain fully liable for servicing their "Pfandbriefe" with the additional investor recourse to the mortgage pool in case of default. The generally low perceived credit risk of the “Pfandbriefe” is further explained by the conservative mortgage haircuts allowed in the mortgage pools which according to the relevant German law is limited to 60% of the sustainable lending value (“Beleihungswert”). This should, however, not be interpreted as a 60% LTV requirement for the respective mortgages.^18^

However, while “Pfandbrief” issuances have a long tradition in Germany and are a very important refinancing instrument for some parts of the German banking sector, in Q4 2019 less than EUR 200 billion worth of German RRE mortgages were part of the respective collateral pools of German banks, representing a refinancing share of approx. 14% of the overall German RRE mortgage market in 2019.^19^ As such, while serving as a trusted and reliable refinancing instrument, the existence of the German “Pfandbrief" market and its strict collateral rules must not be interpreted as a guarantee for the quality of the overall German mortgage market lending standards.

## Empirical literature on mortgage credit risks

There is ample empirical research on credit risks in the US mortgage market driven by the experience of the subprime crisis and supported by the availability of granular mortgage data. Empirical evidence using granular mortgage data for European RRE markets is relatively less voluminous but rapidly growing. Overall, these studies provide general guidance with respect to modeling of the key mortgage credit risk components, i.e. the PD and the LGD.

Regarding the PD, studies of the US mortgage market consider two factors as the main triggers of default: negative house price shocks and income shocks. A large part of the literature for the US can accordingly be interpreted in the light of the “equity” vs “ability to pay” default hypotheses (Jackson and Kaserman, 1980). Generally speaking, a negative impact of RRE prices on the PD can be interpreted as evidence for the equity hypothesis while an unemployment shock effect lends support to the “ability to pay” hypothesis. According to the “equity” hypothesis, borrowers’ default decision will depend solely on the net housing equity value. Such strategic household defaults are modeled for instance by Cocco and Campbell (2004). In contrast to the “equity” hypothesis, the “ability to pay” hypothesis predicts that borrowers will default when they are hit by an income shock that renders their income insufficient to service their mortgage. Using Australian household-level data for the years 2006 and 2010, Read et al. (2014) provide consistent evidence and show that the probability to miss a payment is particularly high for households with relatively high debt service ratios. Fuster and Zafar (2015) show for the US that the delinquency rate drops more than half when cutting the required payment by half.

Findings of numerous studies advocate in favor of the so called “double-trigger” hypothesis. Deng et al. (2000) and Elul et al. (2010) are two studies that give empirical support to the hypothesis that simple option models in the spirit of the “equity” hypothesis are not sufficient to model default rates. The negative relationship between house price changes and PDs might stem from the fact that households with negative income shocks find it easier to avoid personal insolvency by selling their house in a boom market rather than during a bust (compare Schelkle, 2018). The “double-trigger” hypothesis also plays a role in stress-testing models: Djoudad (2012), Dey et al. (2008) and Faruqui et al. (2012) use household micro-data to assess the vulnerability of households in an adverse scenario. Although focusing on debt service variables as the main underlying factor of the propensity to default, housing wealth - or more broadly total wealth - is shown to play a significant role. Other studies use debt-to-income or debt service variables from household surveys to identify vulnerable households and combine these indicators with housing wealth indicators to approximate potential risk exposures (see e.g. Deutsche Bundesbank 2013; Ampudia et al. 2016; Albacete et al. 2014).

Our paper relates to the broader “equity” vs “ability to pay” credit risk literature and sheds further light on the role of institutional features. In general, strategic default decision are less likely to be relevant in Germany given the full recourse character of mortgages. Therefore, house price shocks are unlikely to be the single driving force behind mortgage defaults. This view is confirmed by our empirical model, which correlates mortgage defaults at the German state level not only with house price dynamics but unemployment shocks as well.

Turning to the modeling of LGDs, approaches vary depending on the degree of data granularity and specificities of the considered mortgage markets. Using macro-data to calibrate a model of mortgage losses, Hott (2015) show that loss rates in the US and Switzerland are related to house price level, the LTV ratio of mortgages, interest rates, and the unemployment rate. Various empirical studies using granular mortgage data for European RRE mortgage markets confirm these relationships (see Rodriguez and Trucharte (2007); Dietsch and Welter-Nicol (2014) and Gaffney et al. (2014) for the Spanish, French and Irish housing markets respectively). Furthermore, empirical studies modeling US loss severities indicate the predominant importance of LTV for determining LGDs (e.g. Clauretie and Herzog (1990); Lekkas et al. (1993); Calem and LaCour-Little (2004); Pennington-Cross (2010), and Qi and Yang (2009)). For European countries, similar evidence is provided by studies with different modeling approaches (e.g. Ampudia et al. 2016; Siemsen and Vilsmeier 2017; Hott 2015; Dietsch and Welter-Nicol 2014). Ampudia et al. (2016) find that the LGD at the level of the household is particularly sensitive to the value of the house. Siemsen and Vilsmeier (2017) is one of the few studies looking at the German residential real estate mortgage market. The study assess the impact of a severe decline in house prices on the solvency of German less significant institutions taking the link between LTV and LGD into account. However, lacking sufficient data for the calibration, they derive the meta-dependency between LTV and LGD based on a review of the literature. Focusing on the determinants of the recovery rates of a portfolio of 1,236 defaulted mortgages in Germany, Ingermann et al. (2016) show that collateralization of a loan is the major driver of the recovery rate for retail loans.

As to the LGD component of our model, our approach is linked to the literature underscoring the relevance of LTV for credit risk. Our paper is the fist to present evidence on the impact of historical LTV on the expected losses in RRE mortgage portfolios of German banks.

Further, our paper relates to the stress-testing literature and proposes a risk assessment methodology. Since the global financial crisis, models of mortgage credit risk have gained increasing attention from policy makers and supervisors who rely on them in prudential stress-testing exercises. The purpose of stress tests is to assess the impact of severe macroeconomic and financial shocks on the resilience of individual financial institutions (microprudential stress tests) or entire financial systems (macroprudential stress tests). A number of national authorities have developed stress-testing tools focusing specifically on mortgage credit risk that are either run autonomously (see Albacete et al. 2014 for Austria, Faruqui et al. 2012 for Canada, to name a few) or are integrated in a broader stress-testing framework (e.g. Daniëls et al. 2017 for the Netherlands). Since 2018, the ECB has been applying a macro-micro model created for the purpose of macroprudential top-down stress testing of the banking sector (Budnik et al. 2019; EBA 2021). We contribute to the literature by developing a methodology where modeling of mortgage PDs and LGDs is accurately tailored to the specificities of the domestic housing and mortgage market.

Last but not least, our analysis is related to the growing empirical literature on borrower-based macroprudential measures. Existing papers measure the effects of lending standards on PDs and LGDs indirectly, by linking them to various metrics of household vulnerability reflecting either the liquidity position of households (e.g. Michelangeli and Pietrunti 2014; Gross and Poblaciôn 2017) or both their liquidity and solvency situation (e.g. Albacete and Lindner 2013; Ampudia et al. 2016; Barasinska et al. 2021). We contribute to this new research area by analyzing the role of lending standards in a more straightforward manner by evidencing the direct link between observed payment arrears to LTV, DTI, and DSTI metrics at the household level, and by linking LTVs to the LGDs in banks’ loan portfolios.

To our knowledge, this is the fist study to present an comprehensive assessment of mortgage credit risks in Germany from a microprudential and macroprudential perspective.

## Analytical framework

Our analytical framework is based on three building blocks. First, we estimate a regional Panel Vector Autoregressive (PVAR) model to evidence the macroeconomic link between default rates, house price dynamics and the unemployment rate in Germany. Second, based on the German Panel on Household Finances (PHF), we run microeconometric regressions to investigate the role of debt affordability and LTV ratios on future loan defaults. Third, taking into account the results from the above analyses, we calibrate a structural model to assess systemic risks in the RRE mortgage portfolios of the entire German banking sector.

### Macroeconomic foreclosure rate model

In order to derive the relationship between the foreclosure rate $\Delta \widetilde{FCR}_{t,2020}$ and key macroeconomic variables, we estimate the following PVAR model:

1 $$\begin{aligned} X_{s,t}=\Phi X_{s,t-1} + \epsilon _{s,t} \end{aligned}$$

with $X_{s,t}=[ \ln (FCR_{s,t}), \Delta P_{s,t}, \ln (U_{s,t})]$ and $\epsilon _{s,t}=[ \epsilon ^{\ln FCR}_{s,t}, \epsilon ^{RRE}_{s,t}, \epsilon ^{Unemployment}_{s,t}]$, where $\Delta P_{s,t}$ is the relative price change in state *s* and $\ln (U_{s,t})$ the logarithm of the regional unemployment rate.^20^ The model is estimated with fixed effects which should also capture any time-invariant structural features not modeled directly in the PVAR (compare Section 2.2).

In a second step, we use a standard Cholesky decomposition to identify the underlying shocks and calculate the impulse-response functions and the variance decomposition to derive the short- and medium-term influence of the macroeconomic variables on the foreclosure rate. The expected signs of the impulse responses are shown in Table 1.

**Table 1:** Expected signs of the impulse-response functions for the estimated PVAR model for the (log) foreclosure rates ($\ln (FCR)$), relative house price changes ($\Delta P$) and the (log) unemployment rate ($\ln (U)$)

Impulse/Response	$\ln (FCR)$	$\Delta P$	$\ln (U)$
$\ln (FCR)$	+	?	?
$\Delta P$	–	+	–
$\ln (U)$	+	–	+

### Modeling credit risk at the household level

Furthermore, we analyse the role of lending standards for loan defaults by relying on a logit regression model linking the risk of mortgage arrears to debt characteristics measured at the household level.

2 $$\begin{aligned} Pr(Arrears_h=1) = F(X_h \beta ) \end{aligned}$$

where a binary variable $Arrears_h$ = 1 if household *h* is unable to service debt obligations and $X_h$ denotes the set of observed household and debt characteristics. The aim is to test directly whether lending standards such as LTV, DTI and DSTI or other individual characteristics of households affect the probability of defaulting on loan payments, taking into account households’ liquidity buffers. Finding a significant link between LTV and payment defaults would support the “equity" hypothesis, while significant effects of income- and liquidity related variables would provide evidence in favor of “ability to pay" hypothesis.

### Structural model to assess overall mortgage credit risk

In this section we describe the general framework for assessing overall credit risk in the German mortgage market. As mentioned above, we focus here on the direct risk transmission channel and seek to quantify the potential credit losses in German RRE mortgages to households of German banks. Conceptually, our framework is similar to a classical *EL* model used in stress testing, with the *PD*, *LGD* as well as the exposure at default (*EAD*) being estimated by separate satellite models which condition on the prevailing macroeconomic environment.

Based on the foreclosure rate model described in Section 4.1, we derive dynamic *PD* estimates via the natural link between default, cure and foreclosure rates. Afterwards we simulate *LGD*s based on the uncollateralized part of housing loans which are dynamically updated based on past price movements and amortization payments. Finally, we calculate the amount of outstanding housing loans (*EAD*) based on past mortgage volumes and historical amortization rates (see Online Appendix A.1 for further details).

Applying an adverse economic scenario to our combined *EL* model, we estimate the potential future provisioning needs for the time period 2021-2023 for all German banks with an outstanding RRE mortgage portfolio larger than EUR 5 million and assess the scenarios’ impact on the banks’ capital ratios.

In the following, we explain the different satellite models and calibration assumptions in some more detail while the complete derivation of the model can be found in the online appendix.

#### Estimation of epected mortgage losses

The euro amount of expected losses ($EL_t^j$) for the mortgage portfolio of each bank *j* in year *t* is calculated as the product of the probability of default ($PD_{t}^j$), loss given default ($LGD_{t,T}^{a,s,K}$), and exposure at default ($EAD_{t,T}^{a,j,s,K}$), summed up across all relevant geographical regions *s*, vintages *T*, (initial) amortization rate *a* and LTV buckets *K*,

3 $$\begin{aligned} EL_t^j=\sum \limits _{a,s,T,K} PD_{t}^j \cdot LGD_{t,T}^{a,s,K} \cdot EAD_{t,T}^{a,j,s,K} \end{aligned}$$

As we estimate *PD*s unconditional on LTV buckets *K* (compare Section 4.3.2), we do not model a possible correlation between PDs and LGDs driven by LTVs. While there is substantial empirical evidence from various countries for a positive correlation between the *PD* and *LTV* and, thus, *LGD* (e.g. Elul et al. 2010; Deng et al. 2000; Epley et al. 1996; Kelly and O’Toole 2016; Dietsch and Welter-Nicol 2014; Calhoun and Deng year or Gaffney et al. 2014), cross-country studies do not yield a completely conclusive picture. In fact, they find very different and, mostly, non-linear, patterns in PD-LTV relations. For instance, Gaudêncio et al. (2019) find a positive relations for some EU countries, but also highlight opposing findings with negative relations between default rates and *ILTV* for other EU countries.^21^ In any case, international evidence is not applicable one-to-one to Germany as institutional features are likely to matter. Against this background, our baseline model operates with an assumption of zero correlation between the *PD* and *ILTV*, but we illustrate the potential effects of a positive correlation in Section 6.4.

#### Modeling mortgage default probabilities

Based on the foreclosure rate model described in Section 4.1, we model the *PD* dynamics of the banks’ mortgage portfolio via

4 $$\begin{aligned} \widetilde{PD}_{t}^j= & {} \overline{PD}_{2020} ^j + \Delta \widetilde{PD}_{t,2020} \nonumber \\= & {} \overline{PD}_{2020}^j + \frac{\Delta \widetilde{FCR}_{t,2020}(U,\Delta P)}{1-\omega _{cure}} \end{aligned}$$

where $\overline{PD}_{2020}^j$ is a bank’s average *PD* of its RRE portfolio as reported in COREP for 2020.^22^ Most importantly, $\Delta \widetilde{FCR}_{t,2020}$ is the PVAR forecasted change in the aggregate foreclosure rate between the last observed year and the simulated year *t* based on the underlying macroeconomic scenario.

The term $1-\omega _{cure}$ takes into account that a certain fraction of defaulted mortgages are cured and do not end in an official foreclosure procedure.^23^

#### LGD modeling approach

We model losses conditional on default by the sum of default fixed costs $LGD_{FC}$ and expected losses from foreclosure at time *t*, multiplied by the conditional probability of a defaulted RRE mortgage not being cured $(1-\omega _{cure})$, i.e. being foreclosed. The expected loss from foreclosing a property is negatively related to the recovery value from foreclosures, which in turn depends on the ratio of the current foreclosure price of the property to the outstanding loan amount:

5 $$\begin{aligned} LGD_{t,T}^{a, s,K}=LGD_{FC} + (1-\omega _{cure})\cdot [1-\min (1,\frac{1-\Delta f_{s,t} }{CLTV_{t,T}^{a,s,K}})]. \end{aligned}$$

A detailed derivation of the formula can be found in Online Appendix A.2.

Hence, the expected loss in foreclosure is mainly driven by the current LTV (*CLTV*) and (the time-varying) foreclosure discount $\Delta f_t$. This is in line with Qi and Yang (2009) who show that the current LTV ratio is the single most important LGD determinant. As such, our LGD model is an extension of the model used in Bundesbank Financial Stability Reviews (see Deutsche Bundesbank 2014 and Deutsche Bundesbank 2015) and is very similar in spirit to the approach used by Gaffney et al. (2014) for the Irish mortgage market.

## Data and scenarios

### Data inputs

As outlined before, the datasets underlying the various building blocks of our analyses stem from various sources and their components have different degrees of granularity.

For the estimation of the PVAR we use regional data on annual house price changes, employment and foreclosures for the 16 German federal states for the period 1991-2019. The time series of annual house price changes in the 16 German states $\Delta P_{s,t}$ are based on data provided by bulwiengesa AG since 1991. Before 2004, proxies for state level aggregates are computed based on individual time series for 127 German cities; from 2004 onwards state-level price changes are based on aggregating data for the 401 German districts. The official foreclosure and employment data are obtained from the Federal Statistical Office and the Federal Employment Agency for the time period 1991-2019.^24^ The unemployment rate $U_{i,t}$ in state *i* during year *t* is constructed according to the ILO definition. The foreclosure rate *FCR* is defined as

6 $$\begin{aligned} FCR_{i,t}=\frac{n_{foreclosures,i,t}}{n_{households,i,t} \cdot w_{mortgages,i}} \end{aligned}$$

with $n_{foreclosures,i,t}$ being the official number of initiated foreclosures reported by the Federal Statistical Office in state *i* in year *t*.^25^$n_{households,i,t}$ is the number of respective households and $w_{mortgages,i}$ the share of households with mortgage debt. $n_{households,i,t}$ is estimated as the fraction of total population and the number of persons per household in state *i*. Estimates for the state-specific (albeit time-indifferent) share of households with mortgage debt and the number of persons per household are derived from the PHF.^26^ See Fig. 7 for a historical time series of the average German foreclosure rate.

It should be mentioned that the time period covered by the data used to estimate the macroeconomic sensitivities of foreclosure rates includes one episode of pronounced macroeconomic distress between 2001 and 2005 accompanied by declining nominal house prices and rising unemployment and foreclosure rates. Furthermore, in 1999 private insolvency legislation in Germany was amended to give private persons an easier way to become debt-free if they apply for bankruptcy, which may have contributed to the significant increase in the foreclosure rates observed in the data. Since 2006 the data feature a steady improvement of macroeconomic conditions with steadily declining unemployment and foreclosure rates and, from 2010 on, accelerating house price inflation.

Furthermore, to shed light on the drivers of credit risk from the individual borrowers’ perspective, we extract detailed information on households with mortgage debt from the PHF. The survey collects information on household income and debt service as well as outstanding debt and holdings of real and financial assets. Importantly, the dataset includes information on the amount of the mortgage taken out to finance a residential property and the value of this property at the time of acquisition, thus, allowing us to determine the LTV at origination. Furthermore, households are asked whether they experienced arrears in debt service payments. Descriptive statistics of the PHF dataset can be found in Table 2.^27^ Importantly, the PHF is so far the single source of micro data on the German mortgage market covering information on both the debt arrears and lending standards like LTV and DSTI.

Finally, the analysis of system-wide RRE mortgage losses is built on the borrower statistics on the one hand, containing data on the volume of outstanding mortgages, and the MFI interest rate (MIR) statistics on the other hand, which provide data on volumes and interest rates of new mortgage lending. As laid out previously, we only include RRE mortgages to households in our analyses as risk transmission channels are likely to be different for RRE and CRE loans to enterprises (compare Section 2.1). Although both statistics provide bank-by-bank data, the borrower statistics cover all German banks while the MIR statistics cover only a representative sample of about 240 German banks. The missing data for the rest are imputed based on the procedure described in Online Appendix A.1. The regulatory reporting dataset is then finalized by adding starting point PDs from COREP ITS data for IRB banks with a reference date of December 2020.

Afterwards we supplement this bank-specific dataset with general information on the lending conditions (LTV at origination, amortization rates as well as prepayment rights) from statistical reports of the credit procurement platform Europace.^28^ The platform reports quarterly the distribution of all RRE mortgage loans procured in the respective time span, enabling changes in distribution to be tracked over time. Loans are originated by 750 institutions from all segments of the banking sector (Kreditbanken, Sparkassen, Genossenschaftsbanken, Bausparkassen) but also insurance companies and financial service providers at the end of 2020. With a market coverage of about 33% in 2020, the Europace data are not necessarily representative of the whole banking sector. Nonetheless, the reported LTVs are broadly consistent with other data sources including supervisory data.^29^ Thus, in the absence of bank-specific data on credit lending standards, the distributional data of Europace can be used as a proxy for the distributions of LTV, amortization rates and prepayment rights of an average bank. Effectively, we assign these average distributions to each bank in our sample; the distribution of LTV (and amortization and prepayment rates) in each specific vintage of new loans is therefore identical across banks.

Eventually, we employ regional house price data provided by bulwiengesa AG for the 401 German districts since 2004 to calculate current LTV distributions. Local housing price growth rates are matched to the banks’ credit portfolios, using the information on the banks’ branch network taken from a directory that covers the location of branches of all German banks within Germany.^30^ This allows us to quantify changes in collateral values since loan origination taking the individual regional structure of banks’ loan portfolios and thus region-specific price developments into account. It should be noted that due to the heterogeneity of regional price developments and differences among the banks with respect to loan flows the distribution of current LTVs will - unlike the initial LTV at origination - differ across banks.

**Table 2:** Descriptive statistics of the PHF dataset

	Households without arrears	Households with arrears
Loan to value at origination (ILTV), %	82.92 (40.24)	74.94 (26.90)
Current debt service-to-income (DSTI), %	27.20 (17.55)	29.22 (14.65)
Current debt-to-income (DTI), %	3.11 (2.57)	3.16 (2.75)
Total financial assets, EUR 1,000	85.20 (185.33)	58.90 (81.51)
Total debt, EUR 1,000	154.37 (138.55)	130.65 (126.41)
Disposable income, EUR 1,000	54.81 (42.39)	44.64 (23.36)
Loan age, years	8.80 (7.00)	10.10 (7.10)
Unemployed	0.05 (0.22)	0.10 (0.31)
Diminished employment	0.04 (0.19)	0.15 (0.37)
No. of observations	1000	20

This table reports the means and in parentheses the standard deviation of the PHF household data set used in this study, separately for households without arrears (first column) and with arrears (second column)

### Macroeconomic scenarios

The paths of macroeconomic variables for the assumed scenarios for the forecast period 2021-2023 are presented in Table 3. Our “baseline scenario” assumes that house price growth and unemployment rates remain at the level of 2020. This favorable scenario is used for comparative purposes only and should not be interpreted as the most likely scenario. Our adverse stress scenario assumes a severe macroeconomic downturn with adverse shocks to RRE prices and the labor market. Over the three-year forecast horizon, RRE prices decline by 30% and the unemployment rate goes up by 6pp. Historical data since 1991 suggest that the implied macroeconomic path of the scenario variables corresponds to the 75th percentile for the unemployment rate and exceeds the 99th percentile for RRE prices. The severity of the stress scenario is considerably higher than the last German housing downturn experienced during the late 1990 s and early 2000 s which was characterized by a less steep decline in housing prices but higher unemployment rates.

**Table 3:** Macroeconomic scenarios

		Last	Baseline			Stress		
*This paper*		2020	2021	2022	2023	2021	2022	2023
RRE price growth rate	$\Delta P$	7.0%	7.0%	7.0%	7.0%	0.0%	− 14.0%	− 18.0%
Unemployment rate	*U*	4.1%	4.1%	4.1%	4.1%	7.0%	8.0%	10.0%
*EBA stress test 2021*			2021	2022	2023	2021	2022	2023
RRE price growth rate	$\Delta P$	–	5.4%	4.3%	4.0%	− 2.9%	− 10.1%	− 6.6%
Unemployment rate	*U*	–	4.7%	3.8%	3.5%	6.0%	7.0%	8.4%

This table reports the projected evolution of the RRE price and unemployment path for the years 2021-2023 in Germany used in this paper as well as in the 2021 EBA Stress test. The unemployment rate is based on the ILO definition

To put our analysis in the current context, we compare our scenarios to the macroeconomic scenarios of the EBA 2021 EU-wide stress test (see Table 3) which also took into account the COVID-19 pandemic. Focusing on the adverse scenario, the EBA stress scenario features a cumulative decline of RRE prices by 18.5% and an increase in the unemployment rate to 8.4%. Hence, compared to these projections for the first three years following the COVID-19-induced shock, the adverse scenario in our analysis is still significantly more severe.

## Empirical results for the German mortgage market

In line with our analytical framework, we discuss below our results with respect to the role of macroeconomic and microeconomic default and loss rate drivers as well as the overall expected credit losses in a severely adverse scenario.

### Drivers of mortgage defaults

#### Foreclosure rates and the macroeconomy

**Fig. 5:**
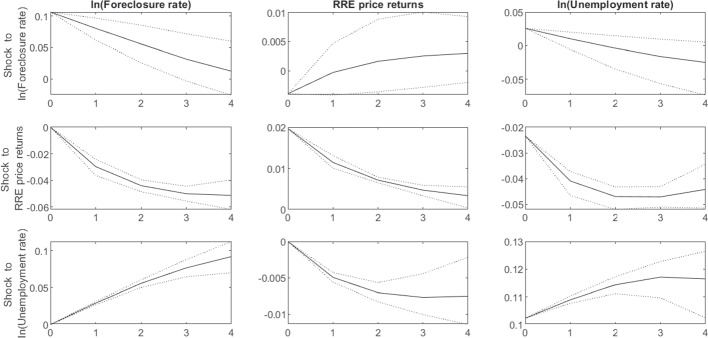
Impulse response functions for foreclosure rate, house price changes and unemployment rate. The plots show responses to shocks of one standard deviation in the variables. Dotted lines indicate the 90% confidence interval

Table 4 displays the regression results while Fig. 5 depicts the impulse response functions from the estimated PVAR based on regional data for the 16 German federal states for the time period 1991-2019.^31^ All impulse responses have the expected signs (compare Table 1) and the effects of RRE prices and the unemployment rate on the foreclosure rates are also statistically highly significant as can be seen in the left-hand column of Fig. 5. Focusing in particular on the response functions of the foreclosure rate, the results confirm that negative price shocks and positive unemployment shocks lead to a positive change in the foreclosure rate.

Furthermore, the effects are not only statistically but also economically significant. According to these estimates, a one standard deviation house price shock increases the foreclosure rate by approx. 5% which is roughly 4bps in absolute values.^32^ A one standard deviation shock to the unemployment rate increases the foreclosure rate by approx. 10% (0.8pp in absolute values). Assuming an average cure rate of $60\%$ in line with our main model calibration, this implies PD increases of $0.1\%$ and $0.2\%$ per standard deviation shock respectively (compare Eq. 4).

**Table 4:** Estimation results for the PVAR model

	$\ln (FCR_t)$	$\Delta P_t$	$\ln (U_t)$
Constant	− 0.019 (0.005)	0.001 (0.001)	− 0.036 (0.005)
$\ln (FCR_{t-1})$	0.661 (0.025)	0.005 (0.005)	− 0.095 (0.025)
$\Delta P_{t-1}$	− 1.142 (0.249)	0.483 (0.047)	− 0.634 (0.253)
$\ln (U_{t-1})$	0.285 (0.023)	− 0.049 (0.004)	1.066 (0.024)

This table reports estimated coefficients for the PVAR model using the (log) foreclosure rates ($\ln (FCR)$), relative house price changes ($\Delta P$) and the (log) unemployment rate ($\ln (U)$) for the 16 German federal states for the time period 1991-2019. Standard errors are in parentheses

**Fig. 6:**
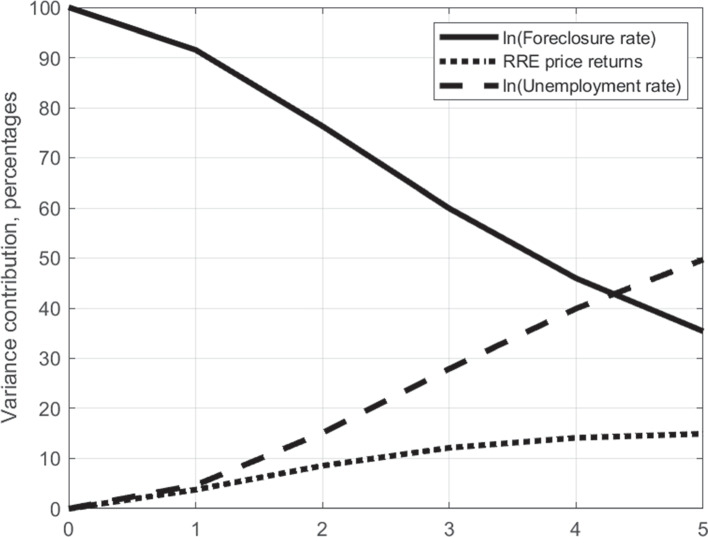
Forecast error variance decomposition of foreclosure rate. Forecast error based on the estimated PVAR model (see Section 4.1)

The higher importance of unemployment shocks for the foreclosure rate can also be seen in the forecast error variance decomposition which is depicted in Fig. 6. While restricted to be zero contemporaneously by the shock identification, the importance of unemployment and RRE price shocks for explaining foreclosure rates in the medium term increases significantly over time. Driven in particular by the increasing impact of the unemployment rate, after four years the two macroeconomic factors explain more than half of the forecast error of the (log) foreclosure rate. As such, the results lend broad support to the validity of the PVAR identification approach and provide initial evidence to corroborate our hypothesis that defaults and foreclosures in Germany are mostly income-driven events.

**Fig. 7:**
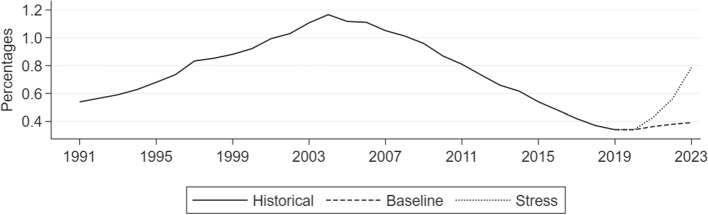
Historical and forecasted aggregate foreclosure rates for Germany over time. The forecasts are based on the estimated PVAR and conditional on the prevailing macroeconomic scenario, baseline (dashed line) or stress (dotted line)

Finally, we forecast the path for the aggregate foreclosure rate conditional on the macroeconomic scenario presented in Table 3, based on the approach by Camba-Mendez (2012).^33^ Fig. 7 depicts the historical time series of the average foreclosure rate for Germany as well as the forecasted values for the simulation period, based on the macroeconomic scenarios. The simulated foreclosure rate strongly increases over the course of the adverse scenario as prices decline and the unemployment rate starts to rise. Finally, we proxy the aggregate PD by the scaled change in the aggregate foreclosure rate (compare Eq. 4). Following the 6pp increase in the unemployment rate in our stress scenario the aggregate PD increases from 1.4% in the last housing boom year to 2.5% at the end of the forecast horizon.^34^

#### The impact of lending standards and household characteristics on mortgage defaults

**Fig. 8:**
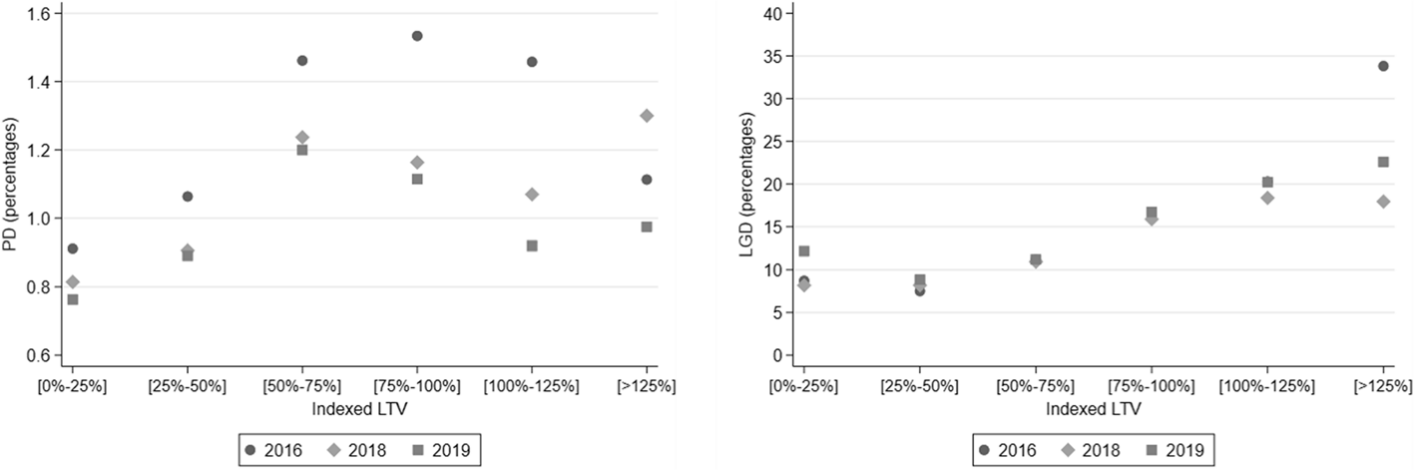
Bank internal volume-weighted PD (left) and LGD (right) estimates by current LTV bucket. Own calculations based on the EBA portfolio benchmarking exercises in 2016, 2018 and 2019

Many of the US-based studies focus on the role of subprime mortgage lending and emphasize the importance of high-LTV loans and negative equity for default decisions (compare Section 3). However, as discussed in Section 2.2, strategic default decisions should be less likely in Germany given the full recourse nature of their mortgages. This appears to be reflected also in PD data from the portfolio benchmarking exercise which do not indicate a monocausal relationship between CLTVs and default rates for the sample of German IRB banks (see left panel of Fig. 8).

To infer further insights on a possible relationship between LTV and other loan and household characteristics, we resort to the household-level data from the German PHF. To isolate the effect of the individual characteristics, we estimate various specifications of Eq. 2. The results of the various model specifications can be found in Table 5.

**Table 5:** Regression results of logit-model for mortgage arrears

	(1)	(2)	(3)	(4)	(5)
ILTV <60%	ref.			ref.	ref.
ILTV 60-80%	$-$0.010 (0.011)			$-$0.012 (0.012)	$-$0.012 (0.012)
ILTV 80-90%	0.008 (0.017)			0.004 (0.018)	0.003 (0.018)
ILTV 90-100%	$-$0.003 (0.013)			$-$0.006 (0.013)	$-$0.008 (0.013)
ILTV >100%	$-$0.011 (0.012)			$-$0.014 (0.012)	$-$0.015 (0.012)
DSTI <20%		ref.		ref.	ref.
DSTI 20-30%		0.022** (0.011)		0.023** (0.011)	0.026** (0.012)
DSTI >30%		0.008 (0.009)		0.009 (0.010)	0.008 (0.010)
Current debt-to-income (DTI),			0.000 (0.002)	$-$0.000 (0.003)	0.000 (0.002)
ln(Total financial assets)					$-$0.003** (0.002)
ln(Disposable income)					$-$0.000 (0.009)
Loan age					0.002 (0.002)
Loan age$^2$					$-$0.000 (0.000)
Unemployed					0.004 (0.016)
Diminished employment					0.023* (0.014)
Survey wave FE	Yes	Yes	Yes	Yes	Yes
Observations	1020	1020	1020	1020	1020
Pseudo-R$^{2}$	0.05	0.06	0.04	0.07	0.11

* p<0.10, ** p<0.05, *** p<0.01. The dependent variable ‘Arrears’ is 1 if a household reports that either one or more debt payments in the past 12 months were made late or not at all, and zero otherwise. The regression is estimated using pooled data of the 2011, 2014 and 2017 waves of the PHF. Reported are the marginal effects evaluated at mean values of continuous variables and at baseline values of the dummy variables. Standard errors are in parentheses

First, our results evidence that the LTV has no statistically significant effect on the probability of arrears in any of the specifications.

Second, in contrast to the LTV, the coefficient for higher DSTI categories (20%-30% and >30%) is positive in all relevant specifications (2), (4) and (5) and is also individually statistically significant for the 20%-30% bucket throughout. In addition to the statistical significance, the effect is also relevant from an economic perspective. The estimated marginal effects suggest that having an DSTI in the 20%-30% (>30%) range increases the probability of arrears by 2.6 (0.8) pp compared to a DSTI<20%. While the coefficient on DSTI >30% is individually not statistically significant, we note that it is only one standard deviation different from the coefficient on DSTI 20%-30%. As a consequence,we use an $\chi ^2$-test to investigate whether the coefficient on DSTI 20%-30% in the regression equation (4) is statistically different from the DSTI >30% coefficient. The $\chi ^2$-statistics of 1.67 at a 19.5% significance level indicates that we cannot reject the equality hypothesis.

Third, DTI was found to be insignificant in all relevant specifications (3 to 5) and irrespective of whether included as a continuous or categorial variable.

Finally, specification (4) of Table 5 shows that financial assets have a statistically and economically highly significant effect on the probability of arrears. The results suggest that having 10% less financial assets increases the probability of arrears by approximately 3pp. Interestingly, only a diminished employment status^35^ appears statistically and economically relevant but not the current employment status. In addition, in contrast to Lambrecht et al. 1997) loan age and disposable income in isolation (i.e. independent of debt service burdens) do not contribute to explaining mortgage defaults in the dataset, either.

Taken together with the PVAR results from Section 6.1.1, these results provide further strong evidence that, due to their full recourse structure, mortgage defaults in Germany are driven mostly by income and liquidity rather than the result of “strategic decisions".

### Drivers of mortgage LGDs

**Fig. 9:**
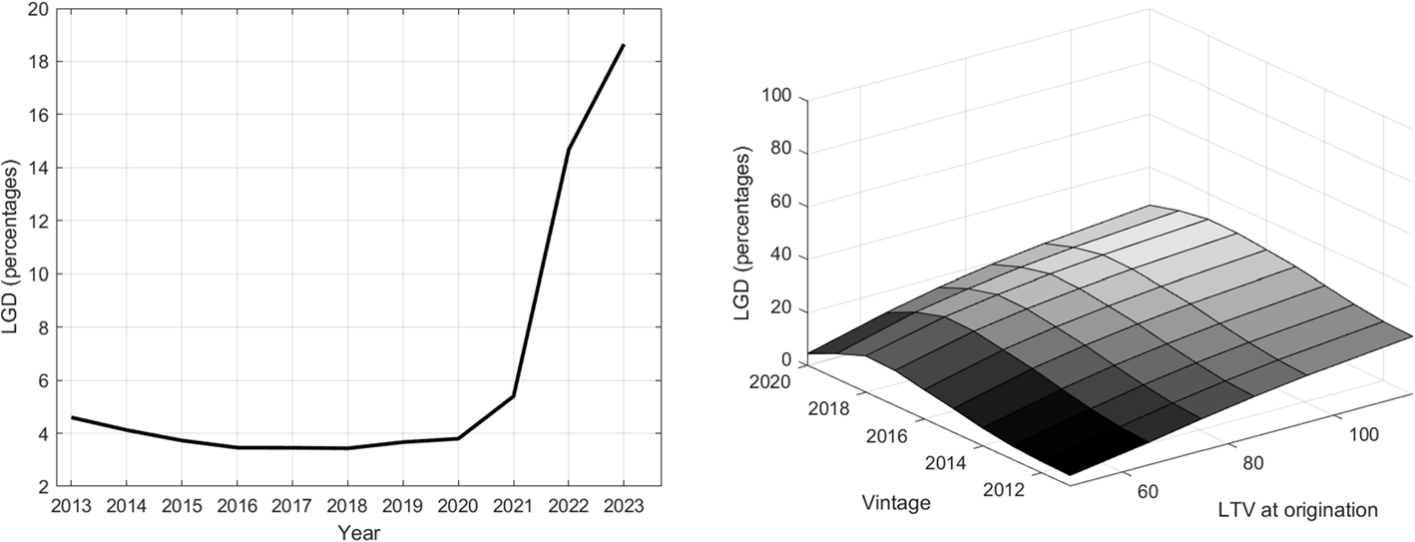
Estimated LGDs over the whole stress scenario (left) and by LTV bucket and mortgage issuance year (vintage) at the end of the stress scenario (right)

The left panel of Fig. 9 shows the evolution of the EAD-weighted average LGD based on our structural model for all banks in sample (compare Eq. 5). Driven by the benign macroeconomic environment and constantly rising housing prices, LGDs are very suppressed at less than 4% at the current juncture but start to rise at the onset of the presumed macroeconomic stress scenario. As a consequence of the cumulative housing price shock of -30% over the three-year horizon, LGDs rise more than fourfold to more than 18% at the end of 2023.

For the interpretation of the LGD in Fig. 9 it is important to keep in mind that the model estimates are based on the mortgages’ current LTVs and can be interpreted as an implicit put option. As a consequence, in an environment of past house price increases, the estimated LGDs are monotonically decreasing and at some point zero for older vintages and for mortgages with lower initial LTVs.^36^

This intuitive and well understood influence of LTVs on LGDs is also reflected in the right panel of Fig. 8 which depicts average LGD estimates per CLTV bucket for the German IRB banks from the EBA benchmarking exercise. The right panel of Fig. 9 shows the monotonic behavior of the LGD with respect to the LTV at origination at the end of the simulation horizon (2023). Generally speaking, higher LTVs at origination are associated with higher LGDs for any given mortgage vintage. The results suggest, in particular, considerably lower credit risk for mortgages with LTVs of less than 80%.

While the monotonic behavior of the LGD with respect to the LTV at origination is economically intuitive, the shape of the LGD curve depends on the underlying macroeconomic scenario. As long as house price changes are positive or zero, the LGD function is decreasing in the age of the mortgage as amortization payments and collateral value inflation outweigh the housing depreciation effect on the CLTV. In our analysis, the 2020 vintage mortgages were effectively assumed to be issued at the peak of the house price boom. Hence, in our simulation, subsequent mortgages are issued at lower prices and, hence, decline less in value until 2023. As such, the shape of the LGD function depends on the relative size of the price changes, depreciation and amortization rates. The curve is positively sloped as long as the amortization rate is smaller than the house price decline and negatively sloped otherwise.

### Overall RRE losses in a severely adverse scenario

Combining the results from individual satellite models presented in Sections 6.1 and 6.2, we now turn to the estimation of potential mortgage credit losses in a severe downside scenario as explained in Section 4.3.

#### Estimated stressed loss rates

**Fig. 10:**
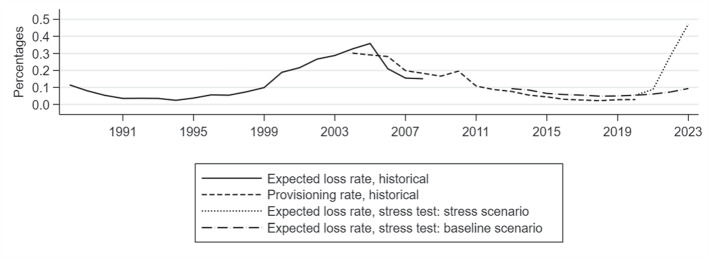
Historical and stress testing loss estimates. Historical expected loss rate until 2008 is based on estimates provided by the German banking association. Historical provisioning rate is derived from the borrower statistics

Figure 10 shows the simulated EAD-weighted average loss rate for the final sample of banks over the entire estimation horizon and puts it into perspective with historical mortgage loss measures. Starting from close to 0.1% in 2013, the simulated loss rate declines by half until 2018-2020 driven by the benign macroeconomic environment, low default rates and increasing collateral values. The simulated evolution is very much in line with historical mortgage loss rates until the end of the observation period (2020).

For the simulated case of a severe macroeconomic deterioration, from 2021 onwards the EL curve is strongly increasing, driven by both rising LGDs from collateral devaluations and increasing PDs. At the end of the simulation period, the average annual loss rate amounts to 0.47% of the total mortgage exposure in the German banking sector. The severity of the stress test losses is considerably higher than in the last housing downturn experienced during the late 1990’s and early 2000’s which was characterized by a less steep decline in housing prices but higher unemployment rates.^37^ In absolute terms, over the entire scenario horizon of three years, the estimated cumulated losses amount to EUR 12 billion.^38^

Furthermore, the expected loss rates in the adverse scenario can be compared to the results from the latest EBA stress test from 2020. For the comparable loan portfolio,^39^ the EBA exercise suggests loss rates of 0.45%, 0.18% and 0.17% for the years 2021 to 2023. While the overall impact is comparable for both analyses (cumulative impact of 0.80% vs 0.85 in this paper), differences in the loss rate patterns are driven by different scenario assumptions and differences in the methodologies.^40^

#### Banks’ own funds under stress

To further assess the severity of stress effects, we measure them in terms of reductions in own funds due to simulated RRE losses.^41^ For the studied banking sample of 1,230 banks, the average Common Equity Tier 1 (CET1) ratio drops over the course of the stress testing from 16.4% to 15.9% due to increased credit losses.^42^ Despite the seemingly muted impact, this is still 0.2pp higher than the comparable impact in the EBA stress test for the largest German banks which, however, feature a disproportionately small share of RRE loans in their portfolios.^43^ However, average values might hide problems at the single-entity level as banks differ with respect to their capital buffers, the size of their respective mortgage portfolios and hence the predicted losses.

As a consequence, we also compare the total losses over the three-year horizon for each individual bank to its respective capital buffers in excess of regulatory requirements at the end of 2020, i.e. the start of the stress period. Specifically, for each bank we calculate its excess capital defined as the difference between the *CET*1 capital and the overall capital requirement for *CET*1. The percentage reduction of the excess capital due to the projected losses over the stress period shows whether a bank is able to absorb these losses without violating its minimum capital requirement.^44^

**Fig. 11:**
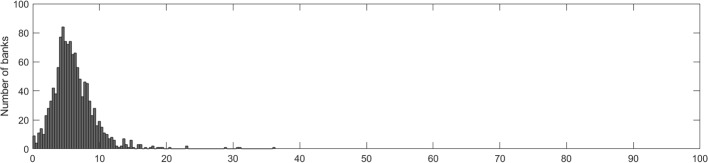
Histogram of estimated excess capital reductions. Excess capital is defined as the difference between the *CET*1 capital and the overall capital requirement (OCR) reported for Q4 2020

Figure 11 shows that for the vast majority of banks the RRE shock would imply an excess capital reduction of 5% to 15%. Only for 11 banks representing a total of EUR 2 billion in CET1 would the housing market shock reduce excess capital by more than 20%, and 2 banks with highly RRE-concentrated loan portfolios would suffer from excess capital reductions of more than 40% but less than 100%.

Hence, the results suggest that the isolated stress effects on capital ratios would be manageable for most of the banks. In addition, they do not concentrate on a few banks but rather spread widely in the German banking sector. It should be noted that, although cross-sectional variation of the stress effects is directly linked to the differences in CLTVs among banks, it does not stem from the initial LTVs as the distribution of initial LTVs is assumed to be identical across banks. Hence, our model captures the differences in the regional structure of banks’ loan portfolios and bank-specific volumes of new lending but is likely to underestimate the dispersion of losses across the banking sector due to differences in lending standards (see also Section 6.4 for a further discussion of this issue).

### The contribution of high LTV mortgages to losses

In Section 6.3.1, we have shown that losses could rise considerably in a downturn scenario with falling RRE prices and rising unemployment. However, a crucial question is to which extent these losses are driven by high-LTV loans as this mortgage segment is generally viewed as a key indicator of the underlying credit risk. For the same reason, LTV limits are often considered to be one of the first macroprudential tools of choice to contain a possible buildup of credit risks in the respective mortgage markets.

As shown in Section 6.2, it is very intuitive and straightforward to show that LGDs are increasing in the CLTV.^45^ However, from a policy perspective the initial LTV at origination (ILTV) is the only available option, since lending decisions can only be influenced at inception. While ceteris paribus higher ILTV loans will also have higher CLTV, the relative importance of the ILTV for the CLTV declines over time as prices fluctuate and loans amortize at different paces.

**Fig. 12:**
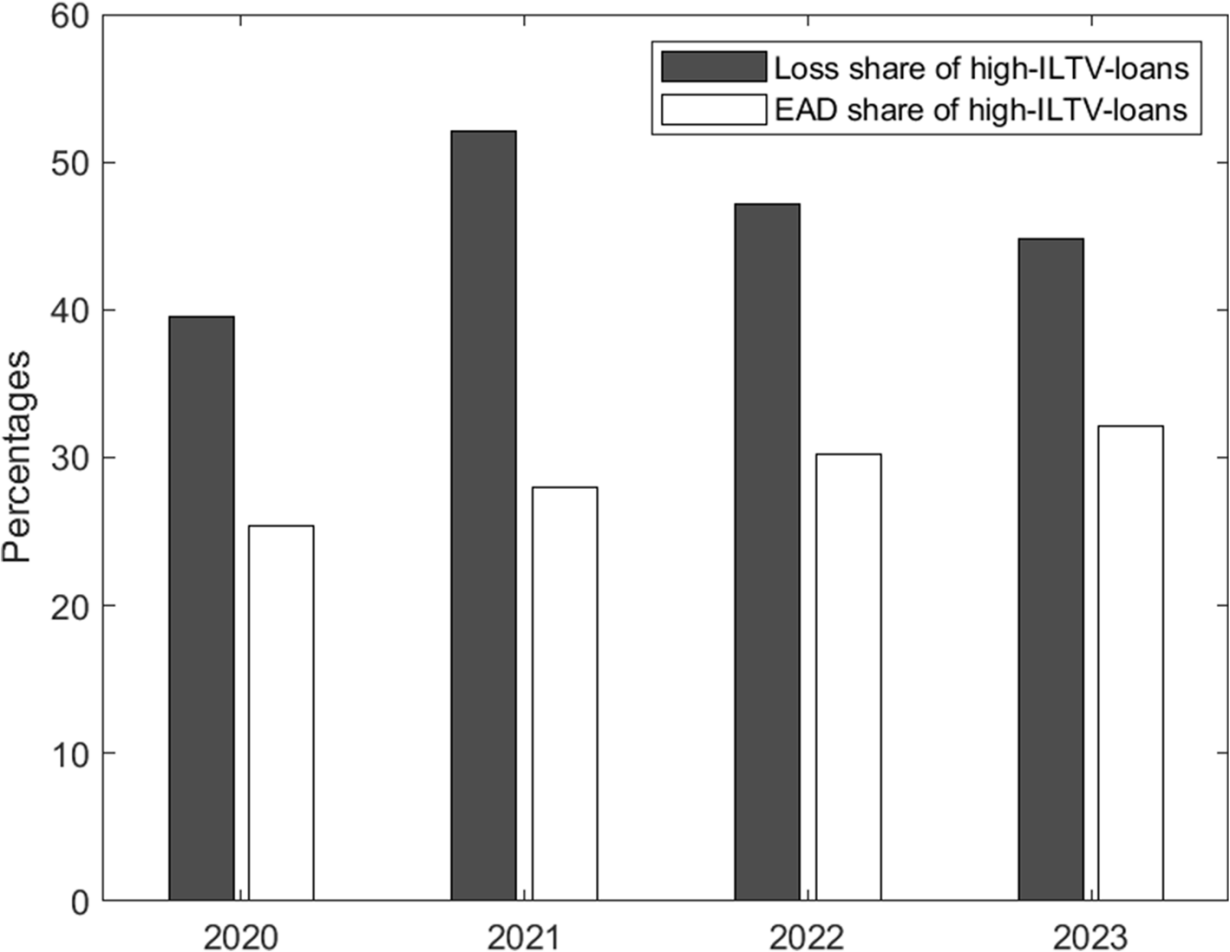
Relative EAD and loss contribution of high-ILTV loans in aggregate portfolio. High-ILTV loans are defined as mortgages with an LTV at origination of more than 90%. Zero correlation between PD and ILTV are assumed

Figure 12 highlights that in 2020 high-ILTV mortgages (as defined by mortgages with an initial LTV of more than 90% at inception) make up roughly one-quarter of the aggregate outstanding mortgage volume in our simulation, in line with the lending data depicted in Fig. 4 left. At the same time, the same high-ILTV mortgages contribute 40% of the simulated losses in 2020 (at the peak of the simulated housing cycle) as most of the other mortgages issued at lower ILTV levels are sufficiently overcollateralised to incur only limited (fixed) costs upon default. Hence, in line with intuition, high ILTV will also make a disproportionately large contribution to losses.

However, Fig. 12 also evidences a second important but, at first sight, less intuitive finding, i.e. the share of losses of high-ILTV loans varies significantly during the cycle and might increase or decrease during stress times. As mentioned above, during periods of increasing housing prices (i.e. until 2020 in our simulation, compare Fig. 9 right panel) only recently granted high-ILTV mortgages will be close enough to negative equity to incur actual losses upon default. As the boom phase ends afterwards, losses on the high-ILTV mortgages will start to mount but other loans will mostly remain in positive equity initially (even after taking into account foreclosure discounts), such that the share of losses of high-ILTV mortgages increases. However, as the housing recession prolongs and deepens, more and more mortgages including low-ILTV mortgages will go into negative equity, outpacing the still increasing losses from high-ILTV loans. While the later still make a disproportionately large contribution to the expected losses, the overall losses are increasingly driven by medium-ILTV losses as the entire market is in turmoil.

At the same time, the results from Section 6.3.1 suggested that the more significant losses only occur in the later year when the crisis deepens and also low-ILTV mortgages exhibit increasing losses. To this end, it seems unlikely that the high-ILTV mortgage segment alone can cause a systemic crisis.

This conclusion might depend, however, on the fact that we model PDs independently of ILTVs. While this assumption is in line with the results presented in Section 6.1.2, it might be that the true PD impact of lending standards (including ILTVs) might only materialize during housing market downturns. In case PDs were positively correlated with ILTVs, estimated loss rates would be structurally underestimated as defaults would be concentrated in high ILTV (i.e. high LGD) buckets.^46^

To illustrate the importance of the effect of different PD-ILTV correlation patterns, we calculate two additional hypothetical EL paths for our stress scenario assuming first a negative and then a positive correlation between PD and ILTV. Based on the international evidence, the correlation is assumed to be non-linear: for ILTVs between 80% to 90% the PD is assumed to be 1.5 times higher (lower) than the PD of ILTVs<80%, while for ILTV>90% the PD is 3 times higher (lower). In addition, in order to facilitate a comparison with the actual data, the PD levels for all ILTV buckets are scaled such that the aggregate average PD remains the same as in our original analysis.

**Fig. 13:**
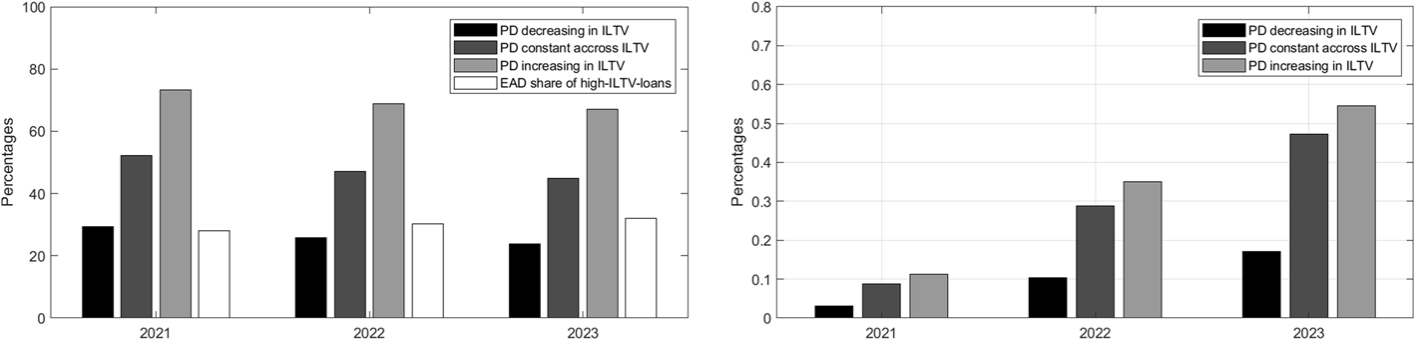
Contribution of high-ILTV loans to losses (left) and estimated loss rate (right) for different assumptions regarding the PD-LTV relationship. High-ILTV loans are defined as mortgages with an LTV at origination of more than 90%

Figure 13 (left) shows the results of these additional simulations. While the scenario-dependent contribution of high-ILTV mortgages is a general finding, the high-ILTV loans contribute at least half of the modeled losses throughout the simulation period in spite of their limited weight in the overall portfolios (approx. 30%). Similarly, under the negative correlation assumption, the relative contribution of high-ILTV mortgages falls below their respective share in the aggregate portfolio. Moreover, the estimated total losses change under different LTV-PD relationship as well (see Fig. 13 right). Compared to the total loss rate estimate of 47 bp in the 3rd scenario year obtained under the zero-correlation assumption, the loss rate is as high as 55 bp given positive correlation and is falls to 17 bp if negative correlation is assumed.

Hence, based on the available data, the results do not support the *a priori* focus on high-LTV loans from a policy perspective, as the relative contribution of those loans might shrink exactly when systemic risks are the highest and crucially depends on the underlying PD correlation.

## Macroprudential perspective and policy considerations

Understanding the underlying drivers of RRE mortgage risk is crucial from a policy perspective as evidenced by the Great Financial Crisis. Yet from a macroprudential point of view, it is important to assess the risks not only from a “banking” perspective but also from a “household” perspective. For the former it is important to assess the banks’ ability to absorb losses under stress which assures a continuous functioning of the financial system to supply the real economy with credit and liquidity. For the latter, the focus is on households’ ability to withstand housing market busts and related macroeconomic growth and income shocks.

Analysing both perspectives – that of households and of banks – is important as the results of our analyses indicate that the policy options suitable to strengthen the resilience under both dimensions might differ.

First, our results show that the direct credit losses in the RRE mortgage portfolios following a general market downturn are sizable but still appear manageable for the banking sector. However, our results on expected losses have to be seen in the context of a broader macroeconomic recession or a financial crisis that goes beyond the housing sector. This is particularly relevant as an isolated residential real estate shock without broader macroeconomic implications is an unlikely scenario. The results from the macroeconomic PVAR analysis suggest that significant spillover effects exist from the housing market on macroeconomic variables such as, for instance, the unemployment rate. Rising unemployment in turn might induce higher loss rates not only for RRE mortgage portfolios but also for other loans to households. Moreover, a housing market downturn is likely to occur in parallel with a significant decline in corporate credit portfolios, in particular for RRE and CRE loans to enterprises and loans to the construction sector in general. This broader macroeconomic view is supported by various empirical studies for the United States^47^ and other OECD countries.^48^

Hence, the overall effects are likely to go beyond the RRE mortgage portfolios and the banking system will face significantly increasing capital needs not only from increasing credit losses but also due to rising risk weights. Therefore, the required level of the capital buffers needed to withstand an adverse RRE scenario should be assessed taking a broader view. Yet from a pure “banking” perspective, broadly based capital-oriented policy measures, like the countercyclical capital buffer, are likely to be the first – because least intrusive – option to strengthen the resilience of the banking system to adverse RRE market shocks.

Second, there is a clear case for complementing capital-oriented policy measures with further borrower-based measures. This is because additional capital requirements will strengthen the resilience of the overall financial systems but do not affect the resilience of indebted households themselves. While banks’ losses are equally determined by PDs and LGDs, households are mainly concerned about avoiding defaults *per se*. Our results support the view that house price corrections alone do not trigger mortgage defaults. Instead, our analysis of household-level data suggests that the probability of default is mostly driven by employment and income-related factors as well as liquidity constraints rather than by LTVs.

In addition, again from a “banking” perspective, our additional analyses in Section 6.4 question whether losses during a severe housing crisis are overwhelmingly driven by high-ILTV mortgages, as long as high-LTV loans are not financing particularly vulnerable households.

As such, our results do not support the *a priori* focus on high LTV loans from a (macroprudential) policy perspective from either the “banking” or “household” perspective. As the inherently associated costs of such policy interventions are likely to increase in the intrusiveness of the individual measure, a combination of instruments aimed at collateralisation (e.g. LTV) and borrower indebtedness (e.g. DTI/DSTI) appears most suitable to address the build-up of systemic risk in the RRE market.

Hence, our results call for an extension of the existing macroprudential toolkit in Germany - currently consisting only of potential LTV caps and an amortization requirement.

Finally, our analysis highlights the importance of a good reference dataset with respect to lending standards for accurate assessment of vulnerabilities of individual banks or certain banking segments to RRE shocks. Data shortcomings may ultimately hinder the assessment of capital needs required to strengthen the resilience of the banking sector. Similarly, further in-depth analyses of driving forces of credit defaults at household level require significant improvements to the reference dataset. Results of empirical studies from other countries can only give indicative guidance as the characteristic institutional features of national mortgage markets are likely to induce asymmetric effects. Seeing the importance of an appropriate reference dataset for the monitoring of risks to financial stability, the German Financial Stability Committee already recommended in 2015 that the Federal Government creates a legal basis for the regular collection of data on housing loans.^49^ Following this recommendation, the Federal Ministry of Finance finally created in February 2021 a legal basis for a regular collection of bank-level data on lending standards.^50^ The data collection is scheduled to start in 2023.

## Conclusion

The German housing market has shown a strong performance over the past decade, including a remarkable resilience even in the light of the current COVID-19 economic recession. On the other hand, elevated property valuations, dynamic credit growth rates and uncertainty regarding the recent evolution of credit lending standards call for a rigorous analysis of the potential risks in the banks’ credit portfolios. The favorable environment for mortgage financing over the last decade may conceal the buildup of vulnerabilities to adverse developments in the housing and labor markets.

In our paper, we analyse the various risk dimensions in the RRE mortgage market. Our PVAR analyses suggest significant links between regional mortgage foreclosure rates and the macroeconomic environment with most of the medium-term variation driven by local unemployment rates. Additional microeconomic household regressions suggest that debt service and affordability metrics as well as available financial buffers can better explain observable default rates than initial or current LTVs. Taken together, these results suggests that mortgage default decisions in Germany appear to be driven mostly by income and liquidity rather than the result of strategic decisions.

Building on the above insights, we estimate expected RRE mortgage credit losses for the entire German banking sector using a structural model. While currently the favorable macroeconomic environment is supporting the low default rates and credit losses, a combined shock to domestic housing prices and labor markets could induce significant additional credit losses in the mortgage portfolios. While our results suggest that the isolated stress effects on capital ratios would be manageable for most of the banks, the cumulative losses could still contribute substantially to the erosion of the risk-bearing capacity of the German banking sector. This is of particular relevance as the historical experience has shown that real estate-related recessions are particularly costly in terms of economic activity. At the same time, our results suggest that high-LTV mortgages are not the sole contributors to systemic risk in Germany as losses are likely to go beyond that particular lending segment during a severe housing crisis. Furthermore, some banks may be more susceptible to housing market shocks than estimated if their lending practices are less conservative than suggested by the available data. The prospective data collection on housing loans in Germany foreseen for 2023 will allow an even more differentiated study of the vulnerability of the banking sector.

Finally from a policy perspective, our results indicate that a combination of capital- and borrower-based macroprudential instruments appears most suitable to tackle buildups of financial stability risks stemming from the RRE market. In particular, borrower-based measures should most likely combine LTV and income-related restrictions. In addition, for a reliable calibration of such instruments, the timely creation of a representative and granular dataset is essential.

## Supplementary Information

Below is the link to the electronic supplementary material.

Supplementary file 1 (pdf 298 KB)

## Footnotes

1 See the public warning of the ESRB on medium-term vulnerabilities in the residential real estate sector in Germany (European Systemic Risk Board 2019).

2 The long time series of the Deutsche Bundesbank is constructed from data provided by bulwiengesa AG (up until 2005), vdpResearch GmbH (2006-2013) and Destatis (from 2014), https://www.bundesbank.de

3 This regional heterogeneity is neither a new phenomenon nor unique to the German housing market. Glaeser et al. (2012) find large regional variations in house prices for the US.

4 Seasonally adjusted. Until 1991, data only for West Germany. Own calculations based on banking statistics, source: https://www.bundesbank.de.

5 Own calculations based on banking statistics, source: https://www.bundesbank.de.

6 Source: Federal Employment Agency, https://www.arbeitsagentur.de.

7 Source: Eurostat, https://ec.europa.eu/eurostat.

8 More details about the German housing market, e.g. property-related taxes and past policy interventions, can be found in Geiger et al. (2016).

9 See German Financial Stability Committee, Recommendation on new instruments for regulating loans for the construction or purchase of residential real estate, 30 June 2015.

10 See Bundesgesetzblatt 2017, No 34, Bonn, 9 June 2017.

11 According to Europace, the share of loans with LTV at origination above 100% in total new lending increased from 5% in 2016 to 20% in 2020. LTV is defined as the share of house purchase price (or the construction costs) financed by a loan. Source: Europace-Baufinanzierungs-Index (EBIx), https://report.europace.de.

12 Own calculations based on LTV distribution reported by of Europace. Uniform distribution of loans within the LTV buckets and a minimum (maximum) LTV of 50% (130%) are assumed.

13 See vdp Spotlight March 2020, Structures of Residential Property Finance 2019 in Germany, https://www.pfandbrief.de.

14 From the banks perspective, however, longer interest rate fixation periods may induce higher interest rate risk in the banking book depending on prepayments and hedging decisions.

15 See The Association for Financial Markets in Europe (AFME): Securitisation Data Report European Structured Finance Q2: 2019, https://www.afme.eu.

16 Including mortgage-backed securities and collateralized mortgage obligations issued by Ginnie Mae, Fannie Mae, and Freddie Mac. See the Securities Industry & Financial Markets Association (SIMFA): US MBS Issuance and Outstanding, https://www.sifma.org.

17 See the Federal Reserve: Mortgage Debt Outstanding, https://www.federalreserve.gov.

18 In fact, each individual mortgage’s loan-to-sustainable value ratio can exceed 60% but the exceeding part of the claim is not eligible for the Pfandbrief coverage pool. For important definitional differences between German loan-to-sustainable lending value (“Beleihungswertauslaeufe") and other standard LTV definitions, visit the website of the “Verband deutscher Pfandbriefbanken" at https://www.pfandbrief.de.

19 Compare transparency figures provided at https://www.pfandbrief.de.

20 We also estimated a specification including the level of FCR. However, this specification yielded a worse fit compared to the specification using $\log {FCR}$.

21 The German mortgage market was not part of this cross-country analysis.

22 As this value is only reported for IRB banks, for banks under the standardized approach we use the average value of all IRB banks.

23 This approximation assumes that the share of defaulted mortgages which are foreclosed remains constant. For a further discussion, see also Online Appendix A.3.

24 The data can be downloaded from the website of the Federal Employment Agency.

25 Strictly speaking, the number of initiated foreclosures reported by the Federal Statistical Office includes all foreclosure procedures of immovable properties, including but not limited to foreclosures of residential real estate. While a breakdown by the type of underlying property is not available, it is most likely that the vast majority of cases refer to residential real estate. Even in the presence of a broader foreclosure definition, the shape and the relative impact of the estimated shocks remain unaffected as long as the ratio between residential real estate and other foreclosures is constant over time.

26 The PHF is a panel survey on household finance and wealth in Germany conducted by the Research Centre of the Deutsche Bundesbank. For details see https://www.bundesbank.de.

27 In particular, we use the following variables from the dataset in our study. *Arrears* is binary variable =1 if households report “*one of the payments made late or not at all*" or “*more than one of the payments made late or not at all*". *Loan-to-value ratio* (LTV) is the ratio of the initial mortgage loan to the value of financed property at loan origination. *Current debt service-to-income ratio* (DSTI) is the ratio of the sum of all debt payments at the time of survey to current disposable household income. *Current debt-to-income ratio* (DTI) is the ratio of debt outstanding at the time of survey relative to current disposable household income. *Financial assets* is the log of all liquid assets excluding sight deposits. *Disposable income* is the sum of incomes of all adult household members. *Loan age* measured in years since loan origination. *Employment* is a binary variable =1 if the household head is unemployed. *Diminished employment* is a binary variable =1 if the household head is in part-time early retirement, low-paid part-time or irregularly employed.

28 See EUROPACE-Baufinanzierungs-Index (EBIx), https://report.europace.de/ebix-etb/europace-ebix. LTV is defined as the share of house purchase price (or the construction costs) financed by a loan.

29 The supervisory data were collected in a one-off survey and are just a snapshot of LTV distribution in 2016-2018 (see Section 2.2). Moreover, for confidentiality reasons, the bank-specific data not available for the research purposes.

30 Hoppenstedt Banken Ortslexikon, as of 2016; the information is further complemented by own research.

31 The panel, however, is not strictly balanced as regional foreclosure data are not available for the early years of the sample for the five eastern states. As a robustness analysis, we repeat the PVAR analysis based on the representative and fully balanced panel county-level price data from 2005 onwards. Quantitatively, the estimated individual impulse response functions are slightly smaller, probably due to the shorter time span which excludes the buildup of the last RRE bubble and the peak of the last crisis.

32 Impact calculated at mean historical foreclosure rate of 80bps.

33 We thank Frieder Mokinski for providing the forecasting code.

34 We checked the robustness of the results by estimating the change in the default probabilities from the employment transition probabilities based on the household survey data from Socio-Economic Panel (SOEP). See Online Appendix A.4 for further details.

35 Diminished employment refers to part-time early retirement, low-paid part-time or irregularly employed.

36 Note, however, that these model estimates are based on averages across all regions and assumed amortization rates. Hence, individual mortgages within each bucket, e.g. with low past price increases and amortization rates, might still be exposed to credit risk.

37 If, instead of the adverse scenario, a favorable scenario with positive housing and job market developments is assumed, our model predicts for the years 2018-2020 continued low expected losses.

38 Assuming constant EAD over the scenario horizon.

39 Retail: Secured by real estate property; non-SME for Germany.

40 In particular, the EBA stress test models credit losses for large German institutions based on an IFRS9 approach, including expected lifetime credit losses for Stage 2 and 3 exposures and a perfect foresight assumption. In contrast, the methodology underlying this paper is more aligned with a national GAAP and the incurred loss principle followed by the overwhelming majority of the rest of the German banking sector.

41 The estimated capital reductions consider only credit losses and no other balance sheet or income effects. Note, that this analysis also ignores capital ratio effects from changes in risk-weighted assets, in particular due to changes in risk weights.

42 Median CET1 ratio drops from 15.7% to 15.3%.

43 CET1 ratio impact for the EBA stress test of (0.29%) was calculated as the change in total provisions for secured Retail Non-SME loans divided by total risk exposure amounts for German banks.

44 As an illustrative example, if a bank has a pre-stress excess capital of EUR 1 billion and losses of EUR 0.3 billion during the stress period, the percentage reduction of excess capital is 30%. A reduction of more than 100% suggests that the remaining capital will not be sufficient to accommodate regulatory requirements.

45 See Section 4.3.3 or right panel of Fig. 8 for actual LGD data from German IRB banks.

46 Our model only captures the implicit correlation between PDs and CLTV induced by common macroeconomic factors, see Section 6.1.1.

47 See Iacoviello (2005).

48 See Iacoviello (2000); Goodhart and Hofmann (2008); Assenmacher-Wesche et al. (2008) and Igan and Loungani (2012).

49 See Recommendation on new instruments for regulating loans for the construction or purchase of residential real estate, June 2015, https://www.bundesfinanzministerium.de.

50 See the Federal Ministry of Finance’s Statutory Order (Finanzstabilitätsdatenerhebungsverordnung) in force since February 3, 2021, available at http://www.gesetze-im-internet.de/finstabdev.
