# Supplementary material for: No Reason to Worry About German Mortgages? An Analysis of Macroeconomic and Individual Drivers of Credit Risk
Source: J Financ Serv Res. 2023 Jun 7:1–31. Online ahead of print. doi: 10.1007/s10693-023-00409-3 (PMC10246538; doi:10.1007/s10693-023-00409-3)
Supplement: Supplementary file 1 — (pdf 298 KB) [file 10693_2023_409_MOESM1_ESM.pdf]

A Appendix: Additional background on the structural model

A.1 EAD modeling approach

The original dataset contains only information on the flow of historical mortgage volumes for the subset of the German banking sector, while they must be forecasted for all banks outside the MIR sample based on the regression results from Equation (A.5).

In principal, we model the EAD as the historical mortgage lending volumes, adjusted for amortizations, prolongations and impairments, i.e.,

$$EAD^{s,K}_{j,t,T} = Lending^{s,K}_{j,T} \cdot net\ outstanding_{j,t,T}. \tag{A.1}$$

However, neither the historical lending volumes nor the currently outstanding share of the loan are known at a sufficiently granular level of disaggregation and must hence be estimated from more highly aggregated data sources.

Generally speaking, the stock of net outstanding mortgages of vintage T is reduced every year by impairments, voluntary down payments, prolongations and refinancings as well as ordinary amortisation payments. In particular, given information on the past RRE impairment rate $Imp_{j,t}$ of bank j in year t, the share of mortgages with a partial prepayment right of up to $x\%$ of their mortgage ($w_{PPP,x,T}$), as well as the share of historical RRE lending with an interest rate fixation period up to one year ($IRFIX1_{j,t}$) or above one year and up to five years ($IRFIX5_{j,t}$), we can approximate the outstanding loan share by:

$$\begin{aligned} net\ \widetilde{outstanding}_{j,t,T} = {} & [1 - \sum_{z=T}^{t} Imp_{j,z}] \cdot [1 - Prob_{PPP} \cdot \sum_{x=0}^{1} (w_{PPP,x,T} \cdot (t-T) \cdot x)] \\ & \cdot [1 - D1_{t,T} \cdot IRFIX1_{j,T} - D5_{t,T} \cdot IRFIX5_{j,T} - D10_{t,T}] \\ & \cdot [\sum_{a} w_{a,T} \cdot AF_{a,i_{j,T},t,T}] \end{aligned} \tag{A.2}$$

where $D1_{t,T}$,$D5_{t,T}$ and $D10_{t,T}$ are dummy variables that are one if the $t-T$ is smaller than one, five or 10 years respectively, or zero otherwise. Implicitly, Equation (A.2) assumes that all loans are refinanced or prolonged at the end of their interest rate fixation period but no later than ten years.[1] Additionally, we include the simplifying assumption that the probability of exercising the partial prepayment option ($Prob_{PPP}$) is independent of the size of the prepayment right x. Furthermore, $AF_{a,i_j,t,T}$ is the theoretically outstanding amount of a vintage T mortgage in t, based on the annuity formula with initial amortization rate a and the bank's average interest rate charged for RRE mortgages $i_{j,T}$ and $w_{a,T}$ is the share of initial amortization rate a in historical lending volumes of vintage T.

[1] By law, German creditors have the right to renegotiate or early repay their loans prematurely after ten years. Given the continuous fall of mortgage interest rates over the last decade, rational behavior would imply that German customers have used this opportunity to secure significantly lower interest rate costs.

The estimation of EADs is further complicated by the fact that disaggregated historical lending data at the regional level are not available. In addition, the only available representative data source on historical RRE lending volumes since 2003, the MIR statistics, only contains data for a representative sample of currently 240 German banks while stock data of RRE mortgages are available for all German banks from the borrower statistics.

As a starting point for estimating the aggregate lending volume for the banks not included in the MIR statistics sample, imagine the following simple stock-flow model:

$$Stock_{j,t} \equiv Stock_{j,t-1} \times (1 - \alpha_{j,t}) + Lending_{j,t} \tag{A.3}$$

where $\alpha_{i,t}$ is the average annual net amortisation rate for RRE mortgages. Note, however, that similar to Equation (A.2) it includes not only regular down payments but also voluntary prepayments and impairments. Furthermore, because the lending aggregate (based on MIR statistics) is gross of loan refinancing and prolongations, $\alpha_{i,t}$ also includes corresponding effects. By simple rearranging of Equation (A.3) we can write:

$$\frac{Lending_{j,t}}{Stock_{j,t-1}} \equiv g_{j,t} + \alpha_{j,t} = M_{j,t} \tag{A.4}$$

with $1 + g_{j,t} \equiv \frac{Stock_{j,t}}{Stock_{j,t-1}}$.

Now, we can estimate $M_{j,t}$ via the following regression based on the MIR statistics bank sample for the time period 2003 to 2017:

$$\frac{Lending_{j,t}}{Stock_{j,t-1}} \equiv M_{j,t} = \gamma \times g_{j,t} + constant + \sum_{i=2}^{I} \beta_i \times D_{j,i} + \sum_{T=2004}^{2017} \beta_T \times D_{t,T} + \epsilon_{j,t} \tag{A.5}$$

with $D_{j,i}$ and $D_{t,T}$ being banking group and time period dummies. This allows us to predict the historical lending volume for the banks outside the MIR statistics sample by:

$$\widetilde{Lending}_{j,T} = \max(\widetilde{M}_{j,T} \cdot Stock_{j,T-1}, 0). \tag{A.6}$$

In the regression estimation, we use the MIR statistics' total new lending definition which includes prolongations (available since 2003) rather than the lending definition without prolongations (available only since 2014). For each bank, monthly lending volumes are aggregated to annual values, annual growth rates are based on end of year figures. Afterwards, we removed missing or irregular data points as well as outliers from the combined MIR and borrower statistics sample. Observations from banks involved in mergers or acquisitions were dropped from the sample. Furthermore, we exclude data points when the increase of the stock was larger than the observed lending volume or when the difference between $M_{j,t}$ and $g_{j,t}$ was larger than 50 percentage points.

Finally, we make the following two simplifying assumptions. First, the initial LTV distribution only depends on the vintage but is the same for all banks and regions. Second, the regional distribution of RRE mortgages for each bank is proportional to its branch network in 2016.[2] Hence, historical lending volumes by region and ILTV can be approximated as

[2] At the time the analysis was completed, information on the branch network for 2017 was not available.

$$\widetilde{Lending}_{j,T}^{s,K} = w_T^K \cdot w_j^s \cdot \widetilde{Lending}_{j,T}, \tag{A.7}$$

where w_T^K is the average share of loans from ILTV bucket K for vintage T and w_j^s is the share of branches of bank j in region s relative to all of the bank's branches.

Table A1 Summary statistics of the main variables used in the EAD modelling approach. The statistics are reported for the historical lending estimation sample based on the Borrower statistics and MIR statistics for the years 2003 to 2020. $Stock_t$ refers to the stock of outstanding residential real estate loans to private households (including self-employed persons). $Lending_t$ denotes new lending gross of loan refinancing and prolongations

	Sample	No. of observations	Mean	Std.
$\Delta Stock_t/Stock_{t-1}(g_t)$	2003–2020	2,647	0.025	0.067
$Lending_t/Stock_{t-1}$	2003–2020	2,647	0.205	0.083

Table A2 shows the respective regression results while summary statistics of the MIR sample can be found in Table A1.

Table A2 Regression results for the historical mortgage lending model for various specifications. The model is estimated based on data from the borrower statistics and MIR statistics for the years 2003 to 2020 where the dependent variable is normalized by the mortgage stock, i.e. $Lending_t/Stock_{t-1}$. Columns (1) and (2) report the results for the specification where lending includes prolongations; columns (3) and (4) show results for true new lending, i.e. excluding prolongations. All four regression specifications include banking group dummies. Standard errors are in parentheses

	Lending incl. prolongations		Lending excl. prolongations	
	(1)	(2)	(3)	(4)
g_t		0.766***		0.819***
		(0.017)		(0.023)
Constant	0.123***	0.151***	0.113***	0.141***
	(0.009)	(0.007)	(0.009)	(0.006)
Banking group dummies	*yes*	*yes*	*yes*	*yes*
Time fixed effects	*yes*	*yes*	*yes*	*yes*
Observations	2647	2647	964	961
R^2	0.307	0.607	0.368	0.720

* p<0.10, ** p<0.05, *** p<0.01

The first two columns of Table A2 report the results of the estimation where new lending also includes prolongations; columns (3) and (4) show results for new lending only, i.e. excluding prolongations. As shown in column (2), the coefficient of the growth rate

($g_{i,t}$) is statistically significant. Its magnitude of 0.766 comes close to the hypothetically expected value of 1.[3] The banking group and time period dummies are statistically significant as well.

As a final step, we predict the total historical mortgage lending volume based on Equation (A.6) for all German banks which are not part of the MIR statistics. In a perfect prediction model, the current outstanding stock of mortgages should be equal to the sum of all net outstanding historical lending flows, i.e.

$$Stock_{j,t} \equiv \sum_K \sum_s \sum_T Lending_{j,T}^{s,K,*} \cdot net\ outstanding_{j,t,T}^{*} \tag{A.8}$$

Hence, we define $EAD\ fit_j$ as the ratio between the actual mortgage stock of a bank and the sum of its model-implied outstanding past lending flows, i.e.,

$$EAD\ fit_j = \frac{Stock_{j,t}}{\sum_K \sum_s \sum_T \widetilde{Lending}_{j,T}^{s,K} \cdot \widetilde{net\ outstanding}_{j,t,T}}, \tag{A.9}$$

which can be interpreted as a measure of goodness of fit. For that reason, we keep in our final banking sample only those banks where $EAD\ fit_j$ is in the range $[0.5; 1.5]$. This condition results in the removal of 16 smaller banks with a share of less than 1% of the overall German mortgage market.[4]

A.2 LGD formula derivation

We model losses conditional on default by the sum of default fixed costs LGD_{FC} and expected losses from foreclosure at time t, multiplied by the conditional probability of a defaulted RRE mortgage not being cured $(1 - \omega_{cure})$, i.e. being foreclosed,

$$LGD_{t,T}^{a,s,K} = LGD_{FC} + (1 - \omega_{cure}) \cdot E(LGD_{t,T}^{a,s,K} | Foreclosure) \tag{A.10}$$

where T denotes the year of loan origination and default fixed costs LGD_{FC} are incurred by the bank irrespective of the workout process.

The expected loss from foreclosing a property is in turn given by:

$$\begin{aligned} E(LGD_{t,T}^{a,s,K} | Foreclosure) &= 1 - Foreclosed\ Recovery\ rate_{t,T}^{a,s,K} \\ &= 1 - \min(1, \frac{p_{s,t} \cdot (1 - \Delta f_{s,t}) \cdot \exp^{(-\delta \cdot (t-T+1))}}{L_{s,T}^{K} \cdot (1 - Amort_{t,T}^{a})}). \end{aligned} \tag{A.11}$$

were $p_{s,t}$ corresponds to the real estate price level in region s at time t and Δf_t equates to the time-varying discount of the property's price on the market value in case of foreclosure, while $\exp^{(-\delta \cdot (t-T+1))}$ is the property's depreciation factor between T and t and $(1 - Amort_{t,T})$ is the part of the loan that has not yet been amortized between T and t.

[3] However, the difference is statistically significant.

[4] Most of the time, the removals are caused by large declines in the reported size of the mortgage portfolio which cannot be explained by ordinary mortgage business and which indicate disinvestments from that business segment and/or reclassification of credit portfolios.

In addition, the following relationship between initial LTVs ($ILTV$) and current LTVs ($CLTV$) holds by definition:

$$CLTV_{t,T}^{a,s,K} \equiv \frac{ILTV_K \cdot (1 - Amort_{t,T}^{a})}{(1 + \Delta P_{s,t,T}) \cdot \exp^{(-\delta \cdot (t-T+1))}} \tag{A.12}$$

where $\Delta P_{s,t,T} = \frac{p_{s,t} - p_{s,T}}{p_{s,T}}$ denotes the cumulative percentage increase in real estate prices in region s between T and t and $ILTV_K \equiv \frac{P_{s,T}}{L_{s,T}^{K}}$.

Combining Equations (A.10-A.12) yields

$$\begin{aligned} LGD_{t,T}^{a,s,K} &= LGD_{FC} + (1 - \omega_{cure}) \cdot E(LGD_{t,T}^{a,s,K} | Foreclosure) \\ &= LGD_{FC} + (1 - \omega_{cure}) \cdot [1 - \min(1, \frac{(1 + \Delta P_{s,t,T}) \cdot (1 - \Delta f_{s,t}) \cdot \exp^{(-\delta \cdot (t-T+1))}}{ILTV_K \cdot (1 - Amort_{t,T}^{a})})] \\ &= LGD_{FC} + (1 - \omega_{cure}) \cdot [1 - \min(1, \frac{1 - \Delta f_{s,t}}{CLTV_{t,T}^{a,s,K}})]. \end{aligned} \tag{A.13}$$

In general, the LGD formula is the same for all banks but average estimated LGDs are different across banks depending on the vintage composition of their portfolios (initial LTVs and amortization rates vary by loan vintage) as well as regional price developments at the location of collateral.

A.3 Calibration parameters

Table A3 shows the parameter choices for the model calibration. The default fixed costs are set at 3%, which is in line with recent EBA portfolio benchmarking LGD data for low LTV mortgages (compare Figure 8). While no representative data source for the cure rate for German mortgages exists, we use available regulatory reported average PDs for IRB banks and average aggregate foreclosure rates over the period 2013 to 2019 to infer implied cure rates according to

$$FCR = PD \cdot (1 - \omega_{cure}) \tag{A.14}$$

$$\omega_{cure} = 1 - \frac{FCR}{PD} \tag{A.15}$$

While we lack sufficient data to estimate its dynamics across the cycle, the figures in Table A4 suggest that the cure rate might be time-varying with the real estate cycle. In an upswing market, more defaulted exposures can be recovered in the free market rather than in official foreclosure procedures, thus, increasing the probability of being cured. Conversely, in a market downturn, the probability of being cured should decrease. Hence, for our calibration we set our through-the-cycle estimate for the cure rate in line with the estimate for the year 2013 ($\omega_{cure} = 0.6$) which should be the least affected by the evolving housing market boom.

In contrast, we assume that the foreclosure discount is time-varying and depends on the general macroeconomic environment and real estate market conditions. In particular, it is

Table A3 Parameter choices for structural model. This table shows the main calibration inputs for the structural model explained in Section 4.3

LGD parameters		
Default fixed costs	LGD_{FC}	3%
Share of defaulted but cured mortgages	ω_{cure}	60%
House price depreciation rate	δ	1.5%
EAD parameters		
Probability of exercising partial prepayment option	$Prob_{PPP}$	40%
Parameters based on PHF survey		
Average share of households with mortgage	$w_{mortgage}$	18%
Average number of persons in households	$n_{persons\,per\,HH}$	2.05

Table A4 Implied cure rate estimates. This table shows average PDs, foreclosure rates and implied cure rates for the time period 2013 to 2019. PDs are estimated from COREP values reported for German IRB retail exposures secured by real estate property of German banks. Foreclosures rates are estimated based foreclosure data from the Federal Statistics office and PHF data according to Eq. 6.Implied ω_{cure} is calculated according to Eq. A.14

	2013	2014	2015	2016	2017	2018	2019	Average
PD	2.0%	2.0%	1.7%	1.7%	1.6%	1.4%	1.4%	1.7%
FCR	0.7%	0.6%	0.5%	0.5%	0.4%	0.4%	0.3%	0.5%
Implied ω_{cure}	67%	70%	69%	71%	73%	74%	75%	71%

reasonable to expect that the discount is smaller during a housing upturn when demand exceeds supply and, in turn, will increase during the downturn when supply outpaces demand. For the US, evidence for disclosure discounts between 0 and 50% are reported by Frame (2010), depending on location and time period, and average discount rates in the range between 10% and 20% are estimated by Clauretie and Daneshvary (2009). Based on these findings, we model the foreclosure discount as a function of current RRE price change, i.e.

$$\Delta f_{i,t} = \max(0, \min(0.5, 0.25 - 2.5 \cdot \Delta p_{i,t})) \tag{A.16}$$

with $\Delta p_{i,t} = \frac{p_{i,t}}{p_{i,t-1}} - 1$ and a natural lower bound at zero and a maximum discount rate of 50% in the most adverse possible market environment. The implied foreclosure discount value of 25% for the years 2006-2011, when average house prices were flat in Germany, corresponds well with the average recovery values of 78% reported by Ingermann et al. (2016) for a portfolio of 1,236 defaulted German properties for the same time period. Finally, the time-invariant annual depreciation rate δ is set to be 1.5%.[5]

[5] According to 2013 OECD estimates, housing depreciation rates (including both structures and land) are generally in the range 1% to 2% per year. The depreciation rate for structures alone is estimated to

A.4 Robustness analysis: PD sensitivities based on SOEP unemployment data

In order to complement the results of the PVAR, we approximate the change in the default probabilities by a matrix of employment transition probabilites based on Socio-Economic Panel (SOEP) data from 1998 to 2012 (see Table A5).[6] Here, we run a series of static and dynamic time series regressions (based on levels and 4-quarter changes) in order to estimate the sensitivity of PD_{SOEP} with respect to the general unemployment rate.

In general, we assume that there two distinct components to default probabilities at the aggregated level. There is a structural time-invariant component, $\overline{PD}_S$, and a cyclical component, PD_C, which is driven by aggregate macroeconomic factors affecting the households income situation, in particular changes in the employment status.

$$PD = \overline{PD}^S + PD^C \tag{A.17}$$

In order to estimate the required macroeconomic sensitivities on the total PD, it is therefore sufficient to estimate the sensitivities of the later component as the structural component is assumed to be time-invariant. In a first step, we estimate the quarterly probability transition matrix for German individuals for becoming employed and unemployed for each quarter based on SOEP data for the time period 1998 to 2012.

Table A5 Average quarterly employment probability transition matrix. Based on SOEP data for the time period 1998 to 2012

	Employed	Unemployed
Employed	0.994	0.006
Unemployed	0.004	0.959

As a second step, we then calculate the one-year forward-looking probability of default PD_{SOEP} as the probability of an individual becoming unemployed some time during the last year and not being employed again within 3 quarters, i.e. remaining unemployed for at least one year:

$$PD_{SOEP} = \sum_{i=0}^{3} p_{EU,t-i} \cdot p_{UU,t+1-i} \cdot p_{UU,t+2-i} \cdot p_{UU,t+3-i} \tag{A.18}$$

with p_{EU} and p_{UU} being the transition probabilities between the states *employed* and *unemployed* and staying unemployed. This yields an average estimate of $PD_{SOEP} = 2.2\%$ for the cyclical component PD_C over the entire time period.

As a final step, we run a series of static and dynamic time series regressions (based on levels and 4-quarter changes) in order to estimate the sensitivity of PD_{SOEP} with respect to the general unemployment rate. According to the results, the PD_{SOEP} estimate increases by 15bps for each percentage point increase in the unemployment rate. This suggests the following law of motion for the estimated default probabilities:

be 1.5% per year.

[6]The SOEP data used in this paper are derived from the Socio-Economic Panel (SOEP) Version 30 (1984-2013) provided by the Deutschen Institut für Wirtschaftsforschung (DIW Berlin). For details on the SOEP Study see Goebel et al. (2018).

$$
\begin{aligned}
PD_t &= PD_{t-1} + \Delta PD_t^C + \Delta PD_t^S && \text{(A.19)}\\
&= PD_{t-1} + \Delta PD_t^C + \epsilon_t^S && \text{(A.20)}\\
&= PD_{t-1} + 0.15\Delta U_t + \epsilon_t^C + \epsilon_t^S && \text{(A.21)}
\end{aligned}
$$

where ϵ_C would include other cyclical (macroeconomic) impact factors. According to this law of motion, a 6pp increase in the unemployment rate in our stress scenario is associated with a 0.88pp increase in the probability of default, which is very close to the predicted unemployment effects of 1pp obtained using the PVAR. Hence, the results of the regression analysis using the SOEP data confirm the findings from the PVAR approach.

References

Clauretie, T. M. and N. Daneshvary (2009). Estimating the house foreclosure discount corrected for spatial price interdependence and endogeneity of marketing time. *Real Estate Economics 37*(1), 43–67.

Frame, W. S. (2010). Estimating the effect of mortgage foreclosures on nearby property values: A critical review of the literature. *Economic Review, Federal Reserve Bank of Atlanta 95*(3), II.

Goebel, J., M. M. Grabka, S. Liebig, M. Kroh, D. Richter, C. Schröder, and J. Schupp (2018). The German Socio-Economic Panel Study (SOEP). *Jahrbücher für Nationalökonomie und Statistik / Journal of Economics and Statistics*.

Ingermann, P.-H., F. Hesse, C. Bélorgey, and A. Pfingsten (2016). The recovery rate for retail and commercial customers in Germany: a look at collateral and its adjusted market values. *Business Research 9*(2), 179–228.
